# Insulin Signaling in Insulin Resistance States and Cancer: A Modeling Analysis

**DOI:** 10.1371/journal.pone.0154415

**Published:** 2016-05-05

**Authors:** Alessandro Bertuzzi, Federica Conte, Geltrude Mingrone, Federico Papa, Serenella Salinari, Carmela Sinisgalli

**Affiliations:** 1 Institute of Systems Analysis and Computer Science “A. Ruberti”, CNR, 00185, Rome, Italy; 2 Department of Computer and System Science, Sapienza University of Rome, 00185, Rome, Italy; 3 Department of Internal Medicine, Catholic University School of Medicine, 00168, Rome, Italy; 4 SYSBIO - Centre of Systems Biology, Milan, Italy; Suzhou University, CHINA

## Abstract

Insulin resistance is the common denominator of several diseases including type 2 diabetes and cancer, and investigating the mechanisms responsible for insulin signaling impairment is of primary importance. A mathematical model of the insulin signaling network (ISN) is proposed and used to investigate the dose-response curves of components of this network. Experimental data of C2C12 myoblasts with phosphatase and tensin homologue (PTEN) suppressed and data of L6 myotubes with induced insulin resistance have been analyzed by the model. We focused particularly on single and double Akt phosphorylation and pointed out insulin signaling changes related to insulin resistance. Moreover, a new characterization of the upstream signaling of the mammalian target of rapamycin complex 2 (mTORC2) is presented. As it is widely recognized that ISN proteins have a crucial role also in cell proliferation and death, the ISN model was linked to a cell population model and applied to data of a cell line of acute myeloid leukemia treated with a mammalian target of rapamycin inhibitor with antitumor activity. The analysis revealed simple relationships among the concentrations of ISN proteins and the parameters of the cell population model that characterize cell cycle progression and cell death.

## Introduction

Insulin resistance represents the common denominator of a series of diseases, including obesity, type 2 diabetes (T2D), metabolic syndrome and cancer. It arises from the impairment of the insulin action, which induces consequently the hyper-secretion of insulin. The main pathways within the insulin signaling network (ISN) are well established [1,2,3], with the serine/threonine protein kinase Akt/PKB and the two mammalian Target Of Rapamycin Complexes (mTORC1 and mTORC2) playing a special role. Akt is phosphorylated on Thr308 by the phosphoinositide-dependent protein kinase-1 (PDK1) and on Ser473 by mTORC2 [4], and the maximal Akt activity is achieved when the molecule is phosphorylated on both residues, allowing the translocation of the insulin-regulated glucose transporters (GLUT4) in muscle and adipose tissue [5,6]. PDK1 and mTORC2 also respond to the activation of the insulin-like growth factor 1 (IGF1) [3].

The kinase cascade through the insulin receptor (IR) up to mTORC1, as well as the mTORC1 activation by amino acids and energy, are clearly assessed [7]. By contrast, the upstream regulation of mTORC2 is not yet well-characterized [8]. The tuberous sclerosis complex 1/2 (TSC1/TSC2) appears to be required for mTORC2 activation [2,9]. However, this view was questioned in a study that reported experimental time courses of several proteins of the ISN under amino acids and insulin stimulation [10]. Interpreting the data by a dynamic model of the network, it was argued that mTORC2 activation pathway may originate from the IR or the insulin receptor substrate-1 (IRS1), possibly via a variant of the phosphatidylinositol 3-kinase (PI3K) [10]. A still different view emerged from experiments in non-diabetic mice both in vivo and in muscle biopsies, and in L6 cells exposed to a medium enriched with proteins secreted by the small intestine of diabetic rats and to serum from insulin resistant humans [11]. This study showed that jejunal factor/s induce insulin resistance and that these factors activate mTORC2, as revealed by an increased value of Ser473 Akt phosphorylation, even in the absence of insulin stimulation. The presence of such intestinal factors is also suggested by the decrease of insulin resistance following bariatric surgery [12].

The mTORC1 substrate p70S6 kinase 1 (S6K1) is involved in the regulation of protein synthesis and the growth of cell size, and active S6K1 inhibits IRS1 in a negative feedback loop [3]. Moreover, the Akt substrate Forkhead box protein O1 (FoxO1) is involved in the regulation of proliferation and apoptosis, so the insulin signaling network has a major role not only in obesity and diabetes but also in cancer [3,13,14].

Following the seminal papers of Wanant and Quon [15] and of Sedaghat et al. [16], several studies have investigated the behavior of the ISN induced by insulin stimulus by developing mathematical models and analyzing the experimental data. Some studies focused on the response to a step increase in extracellular insulin concentration [15,16,17,18,19,20]. In particular, the mathematical model proposed by Kiselyov et al. [17] accounted for both the high and low affinity sites in the two monomers of the insulin receptor. Brännmark et al. [18] studied possible schemes that explain the peculiar behavior observed in the phosphorylation of the insulin receptor and the insulin receptor substrate. More complete dynamical models, supported by the analysis of the time-course of protein concentrations after insulin stimulation, were developed and investigated [10,19,20]. Complex dynamical models were proposed to represent the signaling through the ErbB receptors up to PI3K and Akt, with the aim of exploring the response to an anticancer drug [21], and to model the insulin induced initiation of eukaryotic translation [22].

The dose-response curves, i.e., the steady state concentrations at given insulin levels, were considered in other studies. Giri et al. [23] and Wang [24] studied the behavior of the dose response curves of components of the ISN versus the extracellular insulin concentration, to determine the conditions that produce a hysteresis in the curves as the result of the interactions between negative and positive feedback loops present in the system. Although the experimental time-course of protein concentrations under constant insulin stimulation show that some proteins may not achieve an evident steady state up to 2 hrs [10], the dose-response curves are largely used in the literature to assess the behavior of ISN components at various levels of insulin stimulation and to evaluate the response to perturbing agents and drugs.

Aim of the present study is to investigate the factors that affect the basal protein concentrations and the dose-response curves of the ISN. Using the Michaelis-Menten scheme of chemical reactions, we developed a mathematical model of the network at the steady state, which focuses on the single and double Akt phosphorylation and the upstream signaling of mTORC2. Based on literature data of skeletal muscle lines, we show how the model can represent the effects of gene silencing. The factors that induce insulin resistance are modeled according to the findings in [11]. Improved modeling of Akt and mTOR complexes is also used here to simulate the ISN response in conditions such as TSC2 null and long-term rapamycin treatment. In view of the close relationship between insulin resistance and cancer, mainly due to Akt and mTOR signaling, we combined the insulin signaling model with a cell population model in order to investigate the effects of mTOR inhibitors with antitumor activity on the ISN proteins and on the cell population response.

## Results

**Fig 1:**
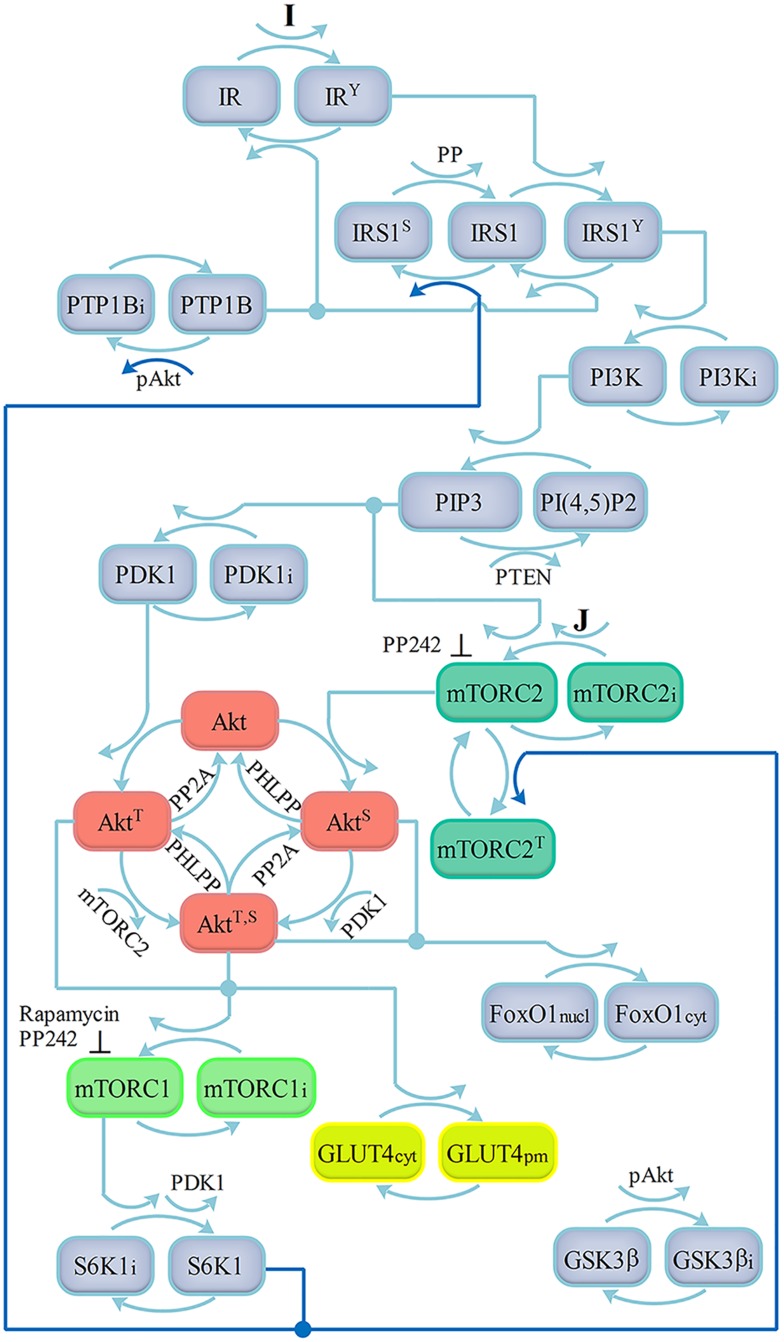
Scheme of the insulin signaling network. Activation by insulin (I) of insulin receptor (IR) catalyzes tyrosine phosphorylation of IRS1. Phosphorylated IRS1 binds the p85 regulatory subunit of PI3K, activating the p110 catalytic subunit. PI3K mediates phosphorylation of PI(4,5)-bisphosphate (PIP2) to PI (3,4,5)-trisphoshpate (PIP3) near plasma membrane (PM) and the phosphatase and tensin homologue (PTEN) dephosphorylates PIP3 back to PIP2. PIP3 recruits Akt and PDK1 to PM, where PDK1 phosphorylates Akt at Thr308 (phosphatase PP2A). mTORC2 is activated by PIP3 and by the factor J, and catalyzes Akt phosphorylation on Ser473 (phosphatase PHLPP). Maximal Akt activity is achieved when the molecule is phosphorylated on both Thr308 and Ser473 residues, allowing translocation of GLUT4 glucose transporters to PM. GSK3 and FoxO1 are direct Akt substrates. Akt also activates mTORC1, which in turn activates S6K1. Activated S6K1 phosphorylates IRS1 and Rictor in negative feedback loops. The positive feedback loop from Akt to PTP1B is also included. Feedback loops are represented by bold lines.

The scheme of our model in Fig 1 is based on the current view of the ISN structure [2,3,14,25]. Since Akt may be independently phosphorylated at Ser473 by mTORC2 and at Thr308 by PDK1 [8], we included all the pathways that lead to the full Akt activation. mTORC2 is assumed to be activated by the phosphatidylinositol 3,4,5-trisphosphate (PIP3), as suggested in [3,26], and by a factor J not dependent on PI3K, which is possibly mediated through the growth factor receptors [11]. The activation of mTORC1 is represented here in a rather simple way, omitting the TSC inhibition by Akt and the consequent mTORC1 activation via the Ras homolog enriched in brain (Rheb). The scheme also contains the direct Akt substrates glycogen synthase kinase 3 (GSK3) and Forkhead box protein O1 (FoxO1). The activated S6K1 phosphorylates IRS1 and Rictor in negative feedback loops [25]. A positive feedback loop from Akt to the protein tyrosine phosphatase 1B (PTP1B) is also included [16]. While the activation pathway to mTORC2 through TSC2 (Huang, Dibble, Matsuzaki, & Manning, 2008) was not considered in view of the results in [10], the present model does not include for simplicity some established pathways of the network, for instance, the IR intracellular pool and the receptor recycling, the TSC2 activation promoted by FoxO1 [27] and the S6K1 activation by GSK3 [28].

The chemical reactions within our ISN model are mostly represented by the classical Michaelis-Menten scheme [29,30], and are discussed in S1 File (Text S1). Several assumptions allowed us to simplify the model. Intracellular localization of proteins (cytosolic vs. membrane-associated), as well as the intracellular trafficking, were neglected. The protein complexes (e.g., mTORC1 and mTORC2) were treated as simple molecular components.

The kinetic equations for the concentrations of the proteins, that also contain the terms of synthesis and degradation of substrates and enzymes, are reported in S1 File (Text S2). As our aim is the analysis of the dose-response curves, we then derived the concentrations of the chemicals at the equilibrium. Under the crucial assumption, as suggested in [31], that the degradation rate constant of a complex enzyme-substrate is negligible compared with the sum of dissociation and catalytic constants, the equilibrium equations take a rather simple form. The ISN model equations in the normalized form used for the analysis of the muscle cell lines C2C12 and L6 are reported in the section Models, Eqs (1)–(16). S1 Table gives the expressions of the parameters in Eqs (1)–(16) in terms of the kinetic parameters of the equations in S1 File (Text S2).

### C2C12 myoblasts

**Fig 2:**
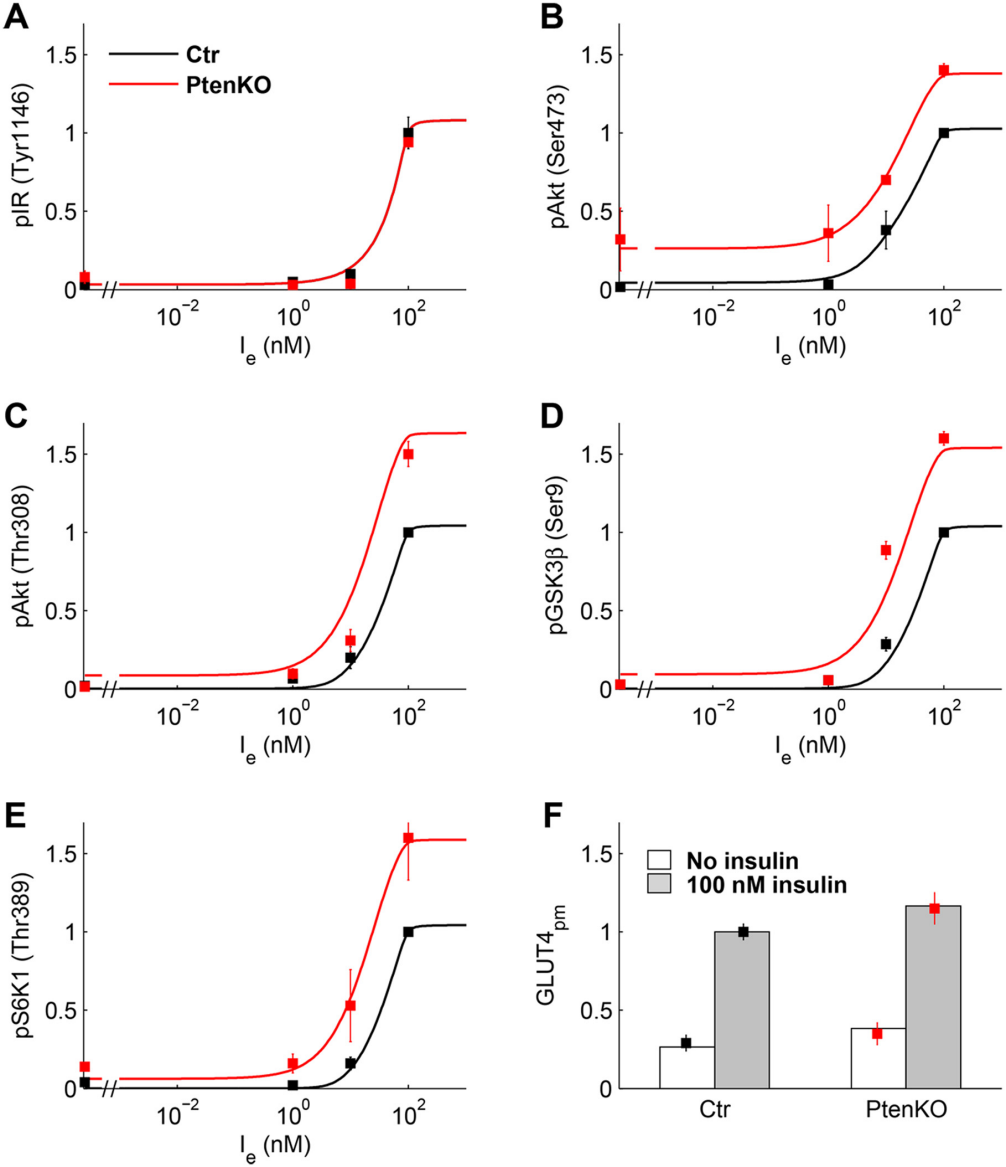
Experimental data of C2C12 myoblast cells and model fitting. Data (mean ± SEM) replotted from Ref [32] for control (black squares) and PTEN-suppressed (red squares) cells. Solid lines are the dose-response curves predicted by the model for control (black) and PTEN-suppressed cells (red). **(A)** Relative pIR(Tyr1146). **(B, C)** Relative pAkt(Ser473) and pAkt(Thr308). **(D)** Relative pGSK3β(Ser9). **(E)** Relative pS6K1(Thr389). **(F)** Relative GLUT4 at PM at zero (white box) and 100 nM (gray box) insulin.

The experimental data in [32] display the normalized phosphorylation levels in C2C12 myoblast cells of IR(Tyr1146), Akt(Ser473) and (Thr308), GSK3β(Ser9), S6K1(Thr389), and AS160(Thr642) at zero insulin and at insulin concentrations of 1, 10, and 100 nM, plus the normalized concentrations of PIP3 and GLUT4_pm_ at zero insulin and at an assigned insulin value. The Authors report the data for control and PTEN-suppressed cells, where PTEN protein concentration was reduced up to 10% of control. We used these data, except those of PIP3 and AS160 that were used for the prediction, to estimate the ISN model parameters. The positive feedback loop from Akt to PTP1B was not included because IR phosphorylation data were similar in control and PTEN-silenced cells (Fig 2 panel A). As done in [20], the normalized experimental data of pAkt(Ser473) were fit by the sum $Akt_{n}^{S}+Akt_{n}^{S,T}$, given by Eqs (10) and (11) in the section Models, because the specific monoclonal antibody is likely to bind Akt phosphorylated on Ser473 irrespective of the presence of the phosphorylated Thr308. Similarly, the data of pAkt(Thr308) were fit by $Akt_{n}^{T}+Akt_{n}^{S,T}$.

Fig 2 shows the data of C2C12 cells, replotted from Ref [32] and used for the estimation of the parameters of Eqs (1)–(16), with *PTEN*_*n*_ in Eq (6) set to one for control and to 0.1 for PTEN-silenced data. Fig 2 also displays the optimal fitting curves computed by the model and S2 Table reports the parameter estimates (with *I*_*e*,0.5_ and *S*_0.5_ given instead of *a*_0_ and *a*_1_). *I*_*e*,0.5_ was found equal to 44.68 nM. As the experimental data are re-normalized to have a unity value at maximal insulin concentration in control, we computed the dose-response curves in Fig 2 according to this constraint.

A subset of model predictions is displayed in S1 Fig. Panel A shows the prediction, obtained by the estimated model, of pAS160(Thr642) together with the data that were not used in the estimation procedure. While the profile of pAS160(Thr642) data was followed rather accurately, the model failed to predict PIP3 concentration data in the PTEN-silenced cells (panel B). We note that if mTORC2 were activated by PI3K instead of PIP3, the model could not adequately fit pAkt(Ser473) data at zero and low insulin in PTEN-silenced cells, nor the prediction of PIP3 concentration data would improve (S1 Fig, panels C, D). The total pAkt (${Akt}_{n}^{T}+{Akt}_{n}^{S}+{Akt}_{n}^{T,S}$) at 100 nM insulin in control is 8.61% of total Akt (S1 Fig panel F) and GLUT4 at the plasma membrane is 48.3% of total GLUT4. These values agree with the model results reported in [16], where pAkt is about 9% of total Akt and surface GLUT4 attains 40% of total GLUT4 after 15 min 100 nM insulin.

S2 Fig shows the sensitivities of protein concentrations upon a ±10% perturbation of the estimated parameters at the extracellular insulin concentration of 44.68 nM. As this concentration equals *I*_*e*,0.5_, the sensitivity to *S*_0.5_ is vanishing. The same occurs for the sensitivities to *a*_12_(below 10^−5^), whereas $a_{13}^{\delta }$ and $a_{17}^{\gamma }$ have small values (S2 Table). The largest positive sensitivities are found for *a*_9_ and *a*_10_, whose values were set equal (see S2 Table) as no data on the phosphorylation of PDK1 and mTORC2 were available. Parameters that directly affect downstream proteins, as mTORC1 and S6K1, also affect the upstream proteins, as IRS1 and PI3K, because of signaling through the negative feedback loop. The opposite behavior of *IRS*1^*Y*^ and *IRS*1^*S*^ is also noted.

As expected, PTEN deletion enhances the insulin response and basal level increased in almost all proteins, according to the negative sensitivity to *a*_8_ of all proteins downstream PTEN, whereas *IRS*1^*Y*^ and PI3K are positively regulated (S2 Fig). In particular, PTEN protein suppression causes an increase in basal Ser473 Akt phosphorylation, which may phosphorylate and deactivate FoxO1 with the possible enhancement of signaling to the pathways that regulate cell proliferation.

### L6 myotubes

**Fig 3:**
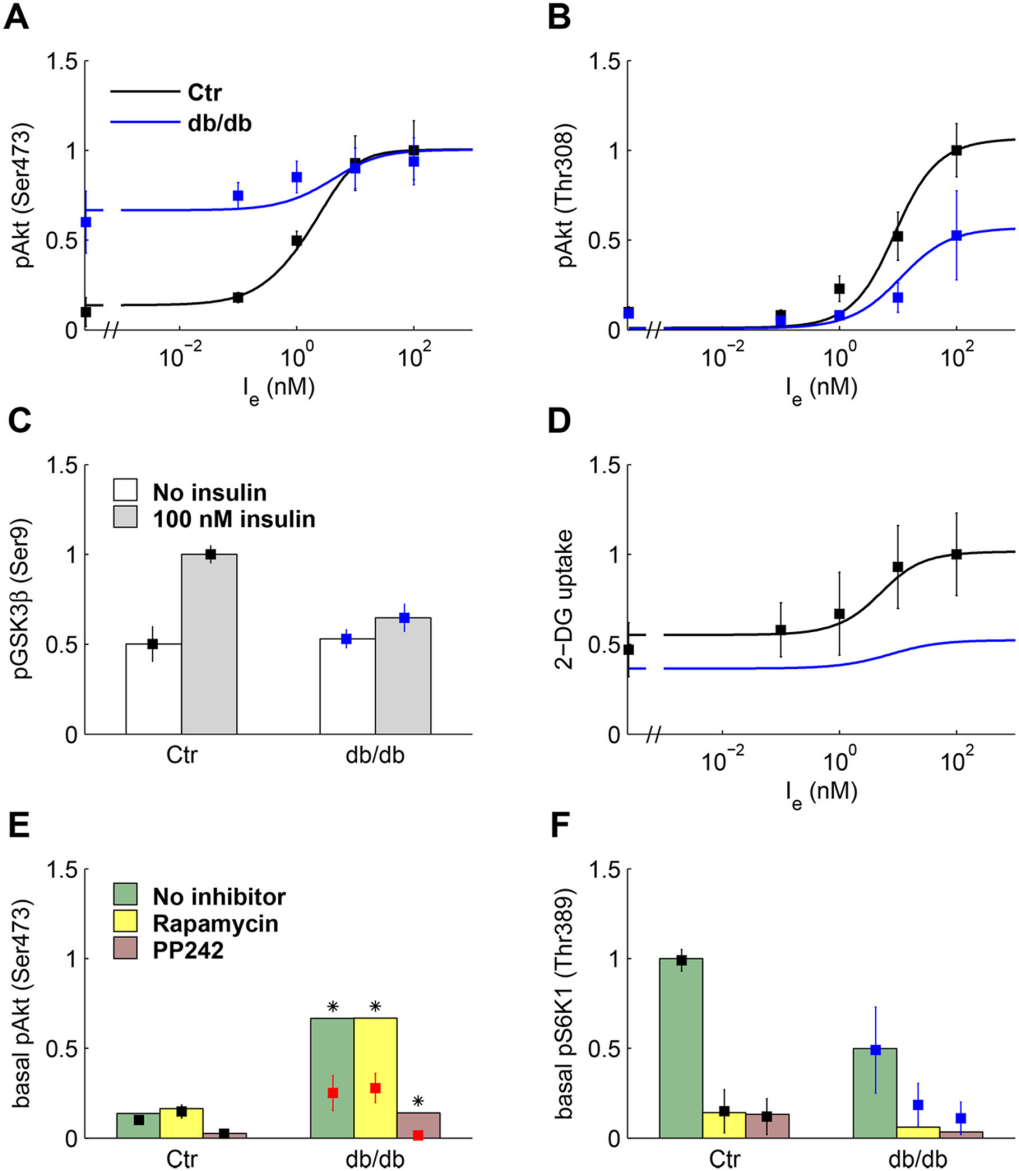
Experimental data of L6 myotubes and model fitting. Data (mean ± SD) replotted from Ref [11] except panel D from Ref [34]. Data (squares) and model fitting (solid lines) plotted in black for control and in blue for cells exposed to conditioned (db/db) medium. **(A, B)** Relative pAkt(Ser473) and pAkt(Thr308). **(C)** Relative pGSK3β(Ser9) at zero (white box) and 100 nM (gray box) insulin. **(D)** Relative 2-DG uptake in rat L6 myoblasts. **(E)** Relative pAkt(Ser473) at zero insulin in control (black) and cells exposed to db/db medium (red), in the absence of inhibition and in cells treated with rapamycin (50 nM) and PP242 (500 nM). The red color indicates that experimental values do not preserve the increase in basal pAkt(Ser473) from control to db/db medium in the absence of inhibition, and asterisks point out that these data were not used in model fitting. Green (no inhibitor), yellow (rapamycin), and pink boxes (PP242) represent model fitting. **(F)** Relative pS6K1(Thr389) at zero insulin in the absence of inhibition and in treated cells (the boxes represent model fitting).

As shown in Fig 3, the data in [11] give the normalized phosphorylation levels in L6 cells of pAkt(Ser473) and (Thr308) at zero insulin and at insulin concentrations of 0.1, 1, 10, and 100 nM. pGSK3β(Ser9) is reported at zero and 100 nM insulin. Moreover, pAkt(Ser473) and pS6K1(Thr389) at zero insulin were measured in the presence of the inhibitors Rapamycin and PP242 that targets both mTOR complexes [33]. The data were obtained in the control medium, enriched by proteins secreted by jejunal mucosa of non-diabetic mice, and in medium enriched by proteins secreted by the mucosa of diabetic mice (denoted in the following as conditioned medium or db/db medium). Based on experiments in non-diabetic mice both in vivo and in muscle biopsies, and in L6 cells exposed to the db/db medium and to serum from insulin resistant humans, it has been hypothesized that jejunal factor/s induce insulin resistance [11]. The factor J that activates mTORC2, see Eq (8), has been included in the model to represent the action of this putative factor.

We fit the experimental data assuming that J has negligible concentration in the control medium and a larger concentration, to be estimated, in the db/db medium. To fit the data obtained in db/db medium, we hypothesized that insulin resistance also increases because of an increased IRS1 degradation due to enhancement of mTORC2 signaling [35]. This negative feedback was represented by suitably tuning the parameters of PI3K. Fig 3 shows the experimental data of L6 cells, replotted from [11] and [34], along with the optimal fitting curves, and S2 Table reports the parameter estimates. We observe that a high value of pAkt(Ser473) at zero insulin, as observed in Fig 3 panel A can only be obtained if mTORC2 is also activated through a signaling pathway independent of PI3K, and if the Thr308 Akt phosphorylation is not required for Ser473 phosphorylation.

Phosphorylation data measured in the experiments with db/db medium are fit with a value of *J* substantially larger compared to control (0.07 vs. 0.001). pAkt(Ser473) at zero insulin is largely increased, but its response to insulin is blunted (Fig 3A). The response of pAkt (Thr 308) and of pGSK3β(Ser9) is also depressed (panels B and C). The 2-DG uptake data reported in Fig 3D were adequately fit by the model. The predicted 2-DG uptake in the presence of db/db medium (panel D) was computed by assuming that the rate constants that regulate GLUT4 translocation to plasma membrane are smaller compared to control [5,6], see S2 Table.

The data measured in the presence of Rapamycin and PP242 are shown Fig 3 panels E-F. The model adequately fits the inhibition of basal (no insulin) pS6K1(Thr389) both in control and db/db medium (panel F). S6K1 inhibition leads in turn, because of attenuated negative feedback, to a decrease in $IRS1_{n}^{S}$ and an increase in $IRS1_{n}^{Y}$ (S2 Fig, panels A-D), thus enhancing insulin signaling. In basal pAkt(Ser473) data (Fig 3, panel E), the poor prediction for cells exposed to db/db medium is caused by experimental variability and the data were not used for model fitting. In cells treated with Rapamycin, the attenuated negative feedback led to an increase of *mTORC*2_*n*_, thus enhancing pAkt. By contrast, PP242 affects Akt phosphorylation at Ser473, so *Akt*^*S*^ and *AkT*^*T*,*S*^ are strongly reduced. A subset of model predictions is displayed in S3 Fig, where panel F gives a 3D representation of the components of pAkt. At 100 nM insulin, total pAkt is 78.7% of total Akt in control. Overall, it appears that the present model provides an adequate fitting of the L6 data.

S4 Fig shows the sensitivities of protein concentrations to the estimated model parameters at the extracellular insulin concentration of 9.69 nM (estimated *I*_*e*,0.5_). The general pattern of the sensitivities for the L6 cells in control and db/db medium is similar to that found for C2C12 cells, confirming that the model is able to represent both types of data. In addition, the sensitivities to $a_{6}^{'}$ and $a_{23}^{'}$ are small according to the small values of estimates whereas, as expected, the sensitivities to the factor J increase in cells exposed to the db/db medium compared to control. The sensitivity to *a*_12_ is small in both C2C12 and L6 cells, suggesting that the negative feedback loop from S6K1 to mTORC2 has a negligible role in these lines.

The L6 cell data were also analyzed in the presence of the positive feedback, with the constant *a*_*P*_ in Eq (4) set to a smaller value for the cells in db/db medium compared to control. The results, however, did not appear to improve on those obtained with the present model.

### Simulations with improved models of Akt and mTOR complexes

Three possible extensions of the models of Akt and mTOR complexes are here considered: sub-cellular Akt localization, mTORC1 activation, and mTORC2 response to rapamycin.

**Fig 4:**
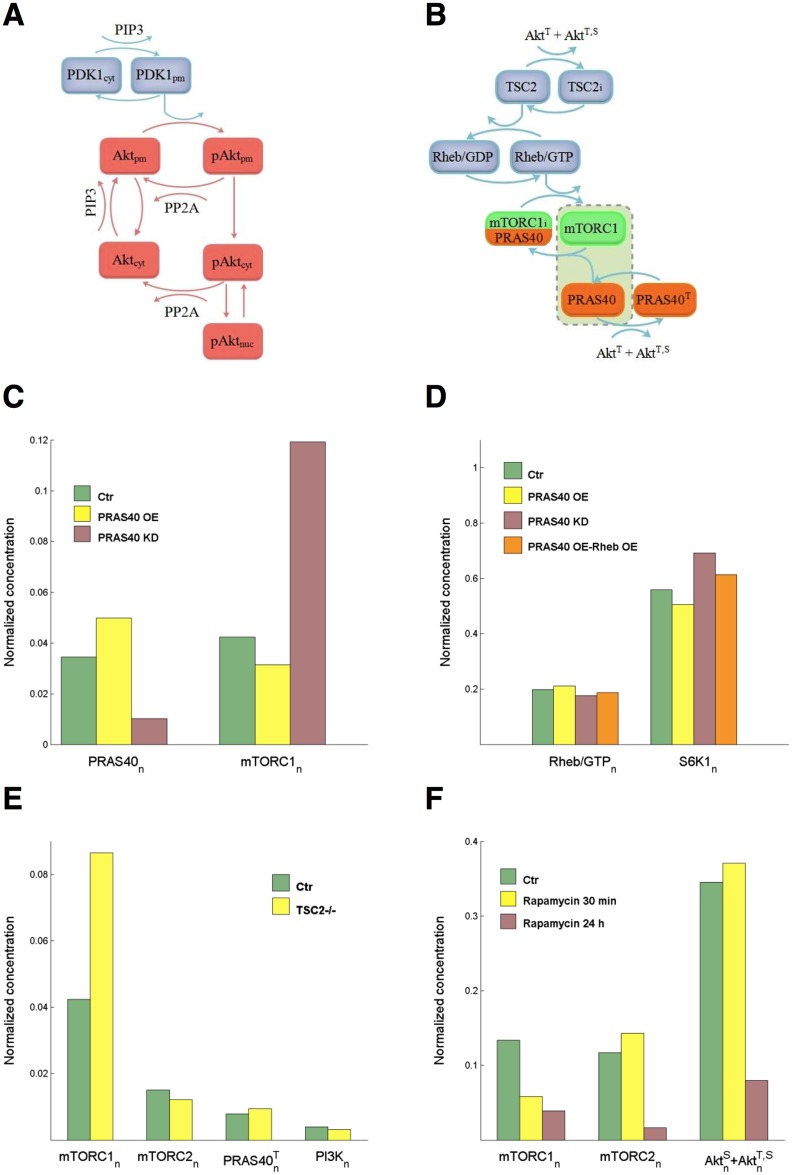
Improved modeling of Akt and mTOR. **(A)** PIP3 recruits PDK1 and Akt to the plasma membrane. At the PM, Akt is phosphorylated by PDK1 and dephosphorylated by PP2A. Transport of not yet phosphorylated Akt from PM back to cytosol is regulated by the rate constant *k*_−13_. Phosphorylated Akt is transported to cytosol (rate constant *k*_*mc*_) where it is dephosphorylated by PP2A or imported into the nucleus (*k*_*cn*_). Export from nucleus is regulated by *k*_*nc*_. (**B**) Phosphorylated Akt inactivates TSC2. Active TSC2 promotes Rheb binding to GDP and TSC2 inactivation stimulates the conversion from Rheb/GDP to active Rheb/GTP, which in turn activates mTORC1. mTORC1 is also inhibited by PRAS40. The box including active mTORC1 and proline-rich Akt substrate of 40 kDa (PRAS40) accounts for reaction (3) in S1 File (Text S3). (**C**) PRAS knockdown (KD: *K*_*mTOR*_ increased tenfold compared to control and *φ* = 0.7, pink boxes) and overexpression (OE: *K*_*mTOR*_ halved compared to control and *φ* = −5/6, yellow boxes) and effect on mTORC1 activation at 1 nM insulin. (**D**) Normalized concentrations Rheb/GTP and of T389 S6K1 at 1 nM insulin with PRAS knockdown (tenfold *b*_*PRAS*_ decrease, pink boxes), PRAS overexpression (twofold *b*_*PRAS*_ increase, yellow boxes), and with both PRAS (twofold *b*_*PRAS*_ increase) and Rheb (fivefold *b*_*Rheb*_ increase) overexpression (orange boxes). (**E**) Normalized protein concentrations in TSC2-null cells at 1 nM insulin. (**F**) Response to short-term and long-term rapamycin treatment of mTORC1 and mTORC2, and effect on Akt phosphorylation at 10 nM insulin. Short- and long-term treatments: *K*_*mTOR*_ in Eq (14) of S1 File (Text S3) set to 0.1 of control. Long-term-treatment: parameters $a_{15}^{\varepsilon },\;a_{19}^{\varepsilon }$ and $a_{19}^{\gamma }$ of Akt Eqs (9)–(11) set to 0.1 of control.

The trafficking of molecules within the cell is regulated by diffusion and active transport, processes that require a complex mathematical treatment based on partial differential equations [36,37]. To give a simplified model of the sub-cellular localization of Akt, we have only considered the Akt phosphorylation at Thr308. Therefore, we have Akt molecules in the cytosolic compartment (Akt_cyt_ and pAkt_cyt_), those located at the PM (Akt_pm_ and pAkt_pm_), and those in the nucleus (pAkt_nuc_). Fig 4 panel A shows a scheme of the model.

The equations of the Akt concentrations are given in S1 File (Text S3) and show that, at the steady state, the three concentrations of phosphorylated Akt tend to be equal provided that *k*_*nc*_ ≅ *k*_*cn*_ and *k*_*mc*_ ≅ *K*_15_ *PP*2*A*. *Akt*_*cyt*_ tends to be equal to *Akt*_*pm*_ provided that $K_{13}^{*}PIP3$ equals *K*_13_ *PDK*1 and *k*_−13_ is much smaller than these two quantities also at low insulin levels. Moreover, *k*_*mc*_ must be much larger than *K*_14_ *PP*2*A*. Although we have no data ensuring that these conditions are satisfied, they guarantee that most of Akt_cyt_ safely reaches PM and is phosphorylated, and that pAkt_pm_ is rapidly translocated from PM to cytosol where it has to phosphorylate several substrates. In these conditions, the Akt model considered in S1 File (Text S1, Text S2) and Eqs (9)–(11), where the sub-cellular localization was disregarded, can be considered an adequate model of Akt kinetics in the steady state. We remark, however, that the transient response of Akt concentrations to a sudden change in the insulin concentration is likely to be different whether the sub-cellular localization is considered or not.

Fig 4 panel B shows the scheme of the improved model of mTORC1 activation. Eqs (12)–(15) in S1 File (Text S3) give the normalized concentrations of the molecular components and literature data provide information on the protein response to Akt. TSC2 phosphorylation level at T1462 has a more than 10-fold increase in HEK-293 cells upon serum stimulation [38]. The increase in PRAS40 phosphorylation level at T246 may range from about 10-fold to 20-fold in isolated skeletal muscle with smaller values in heart, liver and adipose tissue [39]. These data provided constraints in the estimation of the parameters in Eqs (12)–(15), and uniqueness of the estimates was guaranteed by setting *φ* = −0.67 (*b*_*PRAS*_ = 3*b*_*mTORC*1_, i.e., the rate of PRAS synthesis is three times that of the heterotrimer mTOR, raptor, mLST8) and $K_{mTOR}^{\mu }\;=\;10^{-4}$. To estimate the remaining parameters, we took the profiles of $Akt_{n}^{T}+Akt_{n}^{T,S}$ and of *mTORC*1_*n*_ as a function of *I*_*e*_ obtained from the model of L6 cells. The profile of $Akt_{n}^{T}+Akt_{n}^{T,S}$ was used as the input function in (12) and (15) of S1 File (Text S3), and the parameter values that optimally reproduced *mTORC*1_*n*_ profile were as follows: *K*_*TSC*_ = 65.95, *K*_*Rheb*_ = 6.48, *K*_*mTORC*1_ = 7.2·10^−3^, *K*_*PRAS*_ = 16.72.

**Fig 5:**
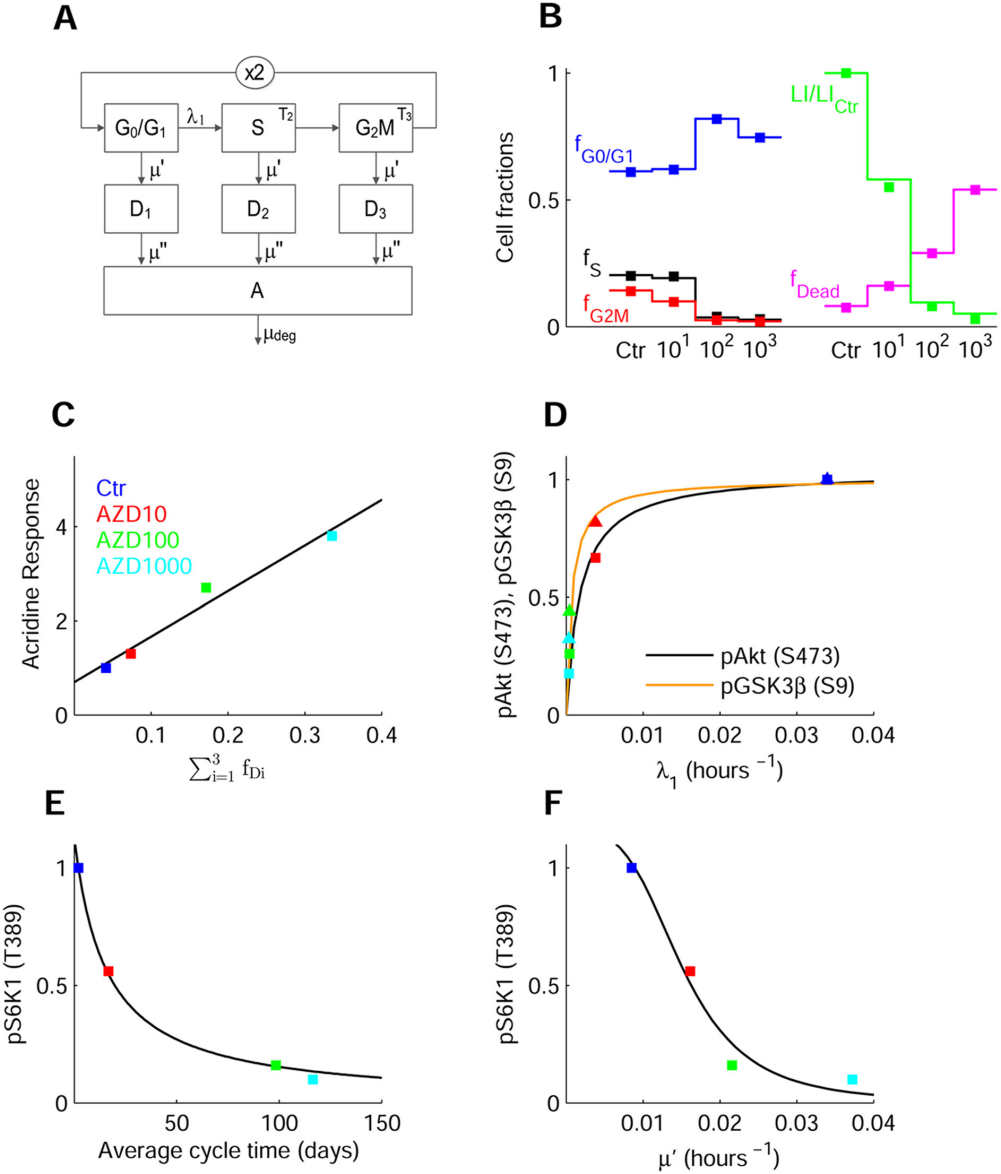
Response of AML cell population to mTOR inhibitor with antitumor activity. (**A**) Scheme of model used for the analysis of AML cell population data in the absence and presence of AZD8055. The blocks represent G0/G1, S and G2M cells, with the ×2 block denoting binary cell division. *λ*_1_ is the rate constant of G1S transition, *T*_2_ and *T*_3_ the transit times in S and G2M phases, and *μ*′ the rate constant of cell loss. D_1_–D_3_ represent cells lost from viable compartments but still measurable, and A the apoptotic bodies and fragments, with *μ*′′ the rate constant of cell fragmentation. (**B**) Data, replotted from Ref [44], of cell fractions in cell cycle phases in control and cells treated with 10, 100, and 1000 nM AZD8055 (closed squares), and model fitting (solid lines). The panel also displays data and fitting of LI normalized to control, and of total fraction of dead cells and fragments. (**C**) Correlation between data of acridine orange staining in A549 cells, replotted from Ref [43], and fraction of dead cells $\sum_{i\;=\;1}^{3}f_{Di}\;$. (**D**) Relationship between the decrease of pAkt(Ser473) (squares) and that of *λ*_1_ at increasing drug concentrations. Fitting line *y* = 1.03*x*/(0.18·10^−2^+*x*), with *y* = pAkt(Ser473) and *x* = *λ*_1_. A similar function fits the relation between GSK3β(Ser9) (triangles) and *λ*_1_. Data are normalized to control and represented with the color code in panel C. (**E**) Decrease of normalized pS6K1(Thr389) with drug concentration and relation with the average cell cycle time *T*_*c*_ = 1/*λ*_1_+*T*_2_+*T*_3_ predicted by the cell population model. Fitting line *y* = 17.71/(15.61+*x*). (**F**) Decrease of pS6K1(Thr389) with the drug concentration and relation with the parameter *μ*′ predicted by cell population model. Fitting line *y* = 3.63·10^−7^/(3.15·10^−7^+*x*^3.57^).

In the simulations presented in Fig 4, panels C-F, we used the complete ISN model of Eqs (1)–(16) with Eq (14) of *mTORC*1_*n*_ replaced by Eqs (12)–(15) of S1 File (Text S3). The loss of PRAS40 expression was represented in the model by a tenfold decrease of *b*_*PRAS*_ compared to control, with the consequent changes of *K*_*mTOR*_, $K_{mTOR}^{\mu }$ and *φ* in (14)-(15) of S1 File (Text S3). Fig 4C shows the decrease of *PRAS*40_*n*_ and the increase of *mTORC*1_*n*_ in this condition, compared to control. By contrast, PRAS40 overexpression with a twofold increase in *b*_*PRAS*_ inhibits mTORC1 activation. In Fig 4D, the normalized concentrations of T389 S6K1 under PRAS knockdown and overexpression (pink and yellow boxes, respectively) follow the response of *mTORC*1_*n*_ shown in panel C. Under both PRAS and Rheb overexpression (orange boxes), mTORC1 and S6K1 are no longer inhibited compared to control because GTP-loaded Rheb overcomes the PRAS40-mediated mTORC1 inhibition, as pointed out in [38] and predicted by Eq (14) in S1 File (Text S3). The results of the simulations in panel D appear to agree, qualitatively at least, with the increased T389 S6K1 phosphorylation shown by the blots in Fig 4E (HEK293E cells) and Fig 5E (MEFs and HT-29 colon cancer cells) of [40].

To analyze the effect of chronic mTORC1 activation, we represented the ISN signaling in TSC2-null cells by setting *b*_*TSC*_ and thus *K*_*Rheb*_ to zero. Eq (13) of S1 File (Text S3) shows that *Rheb*/*GTP*_*n*_ attains its (maximal) unity value and mTORC1 concentration increases compared to control (Fig 4E). Because of the enhanced negative feedback, PI3K, mTORC2 and Akt phosphorylated at S473 are downregulated. A highly decreased PI3K concentration compared to control, as found in TSC2 null MEFs [41], was not obtained. A marked decrement might be obtained from the present ISN model by imposing different values of the parameters, and mainly by values of *a*_4_ larger than the value actually estimated in the L6 cells, so to increase the extent of the negative feedback.

A model of the response of mTORC2 to long-term rapamycin treatment is given in S1 File (Text S3). In the simulation of the response to long-term treatment (Fig 4F), the production rate *b*_*mTORC*2_ of mTORC2 was set to 0.1 of control level to account for a value of *α* (the cell-type specific parameter) close to the unity, according to Eq (19) of S1 File (Text S3). The response of mTORC1, both in the short-term and the prolonged treatment, was instead represented by a tenfold decrease of the catalytic constant *k*_3*c*_ (and thus of *K*_*mTOR*_) in (14) of S1 File (Text S3), as done in the analysis of the data of L6 cells. Compared to control, short-term treatment inhibits mTORC1, but enhances mTORC2 due to the downregulation of negative feedback. By contrast, the prolonged treatment strongly inhibits mTORC2 because rapamycin/FKBP12 binds to newly synthesized mTOR and the formation of mTOR complex is prevented. As *b*_*mTORC*2_ appears in the parameters $a_{15}^{\varepsilon },\;a_{19}^{\varepsilon }$ and $a_{19}^{\gamma }$ of Akt Eqs (9)–(11), mTORC2 inhibition causes a decrement of S473 as well as of double Akt phosphorylation and, as a consequence, also mTORC1 is further downregulated. The simulation results agree qualitatively with the reduced S473 Akt phosphorylation shown by the blots in Fig 2 of [42] for cells highly sensitive to rapamycin, as the PC3 cells.

### Response to mTOR inhibitors with antitumor activity

The response of the ISN and of an AML (acute myeloid leukemia) cell population to mTOR inhibitors is studied here with reference to the dual ATP-competitive mTOR inhibitor AZD8055 [43,44]. The study in [44] reports data of Akt and mTOR signaling pathways, together with data that demonstrate the anticancer activity of the drug. Data include the inhibition of p70S6K(Thr389) and pAkt(Ser473) in the MV4-11 human AML cell line, in untreated cells and at drug concentrations of 10, 100 and 1000 nM. The Authors also report the fractions of cells in cell-cycle phases and the apoptotic fragments obtained by propidium iodide (PI) staining and flow cytometry, the data of [^3^H]thymidine incorporation (labeling index, LI), the fractions of annexin V and PI-positive cells, and the *in vivo* effect of the drug in mice bearing MV4-11 xenografts.

The cell population response is represented by the mathematical model of Eqs (17)–(26), described in the section Models, which is similar to the model proposed in [45] and is depicted by the block scheme of Fig 5 panel A. The model parameters, to be estimated from the data in control and in the treated populations, are the rate constant *α* of exponential growth (or decline) of the population, the rate constant *λ*_1_ of the transition G1 S, the transit times *T*_2_ and *T*_3_ in S and G2M, and the cell loss rate constants *μ*′ and *μ*′′. The estimates, reported in S3 Table, show the effects of the different drug concentrations on the cell population kinetics.

Fig 5B displays data and model fitting of the cell fractions in cycle phases, showing the cell accumulation in G0/G1 and the depletion of S and G2M in the treated populations. The same panel also displays data and model predictions of the LI and of the total fraction of dead cells and fragments. The rate constant *λ*_1_ exhibits a marked concentration-dependent decrement, whereas the transit times in S and G2M, and the rate constant *μ*′ of loss from the viable compartment, increase (S3 Table). Accordingly, *α* (population doubling time ln2/*α* = 3.86 days in control) turns out to be negative in the treated populations (halving times 2.036, 1.364, and 0.783 days at 10, 100, and 1000 nM), confirming that a major factor that inhibits cell proliferation is the block of cells in the G0/G1 phase [46]. There is an intricate interplay between autophagia and apoptosis [47]. We did not represent these pathways, hence the simple model used cannot relate the loss parameters *μ*′ and *μ*′′ to autophagia or apoptosis. However, as depicted by Fig 5C, the fraction of dead cells $\sum_{i\;=\;1}^{3}f_{Di}$ nicely correlates with the increase of acridine orange staining reported in [43] for a different cell line, suggesting that *μ*′ might mainly be related to cell death caused by autophagia.

To correlate the kinetics of the AML cell population with the response to AZD8055 of the ISN, we accounted for the constitutive activation of PI3K/Akt signaling, frequently found in AML [48], representing this activation by an “equivalent” insulin signal. To fit the pS6K1(Thr389) inhibition profile in the MV4-11 human AML cell line in [44], we estimated the parameters that regulate mTORC1 and mTORC2 inhibition (*a*_10_, *a*_11_, and *a*_24_ in Eqs (8) and (14), keeping the other parameters to values estimated for L6 cells (S5 Fig panel B).

A simple nonlinear monotonic function provides a good fit of the relation between the value of pAkt(Ser473), predicted by the insulin signaling model, and the population model parameter *λ*_1_(Fig 5D), showing how these two quantities are reduced as drug concentration increases and how the extent of the block of G1S transition is related to mTORC2 inhibition. Similar functions also fit the relations between *λ*_1_ and pGSK3β(Ser9), cytosolic FoxO1 and pS6K1(Thr389) (Fig 5D and S5 Fig panel A). Panel E depicts the relationship between the average cell cycle time, an index of the rate of protein synthesis, and pS6K1(Thr389). In panel F, the model predicted pS6K1(Thr389) is plotted versus *μ*′, showing how mTORC1 inhibition is also related with the increment of cell loss. S5 Fig panel C reports data and model fitting of pAkt(Ser473) as a function of the dose, compared with data in MDA-MB-468 cells [43], and S5 Fig panel D represents the ratio cytosolic/nuclear FoxO1 predicted by Eq (12) along with data in MDA-MB-468 cells [43].

Overall, the above findings agree with the notion that mTORC2 inhibition activates cyclins D1-D2 via Akt(Ser473) and FoxO1 inhibition, and that mTORC1 inhibition activates autophagy [3]. Rapamycin derivatives were indeed found to inhibit both mTOR complexes and decrease the levels of *CCND1* and *CCND2* in AML [49].

## Discussion

The ISN scheme here considered is based on a consolidated view that emerges from recent literature. The present model concentrates particularly on single and double Akt phosphorylation because recent studies [50] have shown that Akt activity is maintained almost unaltered when it is phosphorylated only on Thr308, while Ser473 phosphorylation seems to play an independent role in both insulin resistance and cancer. That Akt can accomplish its enzymatic function without undergoing Ser473 phosphorylation is demonstrated by the finding that muscle-specific *rictor* KO mice simply present with a moderately decreased insulin-stimulated glucose uptake and glucose intolerance [50], but not diabetes. Thr308 Akt phosphorylation is in fact able to activate GLUT4 translocation and is sufficient to mediate the phosphorylation of glycogen synthase kinase 3.

Our model shares a common ground with models previously described in the literature, but it differs in several ways. Specifically, in [10], [16] and [20] the time course of a dynamical model response to an insulin pulse was provided, whereas model equations in our study are solved at the steady state, giving the dose response curves. The present ISN model partly simplifies and partly extends the model in [16] by accounting for both Ser473 and Thr308 pAkt, GSK3β and FoxO1, the complexes mTORC1 and mTORC2, and the mTORC1 substrate S6K1. The final set of equations at the steady state, Eqs (1)–(16), got a simple and clearly readable form by neglecting the degradation of the complexes. We stress that a scheme where Akt can be independently phosphorylated at Thr308 and Ser473 residues, and where both sites can lead to complete Akt activation, has not, to our knowledge, been previously considered. Moreover, the upstream mTORC2 signaling depends on two independent pathways mediated by PIP3 and by a signal possibly related to activation of IGF1 receptor [11]. The parameter estimates reported in S2 Table are rather different between C2C12 and L6 cells, which however is not surprising since C2C12 is a line of mouse myoblasts whereas L6 is a line of rat myotubes.

To fit the data from the L6 cells exposed to db/db medium [11], the following changes in model parameters were done compared to control values. 1) the factor J much larger for cells in db/db medium than in control represents the effect on the ISN of an external factor possibly of intestinal origin. 2) *a*_6_ and $a_{6}^{'}$ smaller than in control represent in a simplified way a negative feedback on IRS1 related to mTORC2 hyperactivation. 3) The putative GSK3β sequestration ($a_{23}^{'}>0$). 4) The decrease of *a*_28_ and *a*_29_ in Eq (16) for GLUT4 represents a diminished capacity of GLUT4 vesicles to reach plasma membrane. Notably, changes similar to those induced on ISN by db/db medium were also induced by serum from insulin resistant humans compared to serum from control subjects [11].

Comparison of the results obtained here by analyzing the data from L6 cells exposed to the db/db medium with those presented in [20] suggests the following observations. The large pAkt(Ser473) level observed at zero insulin in cells exposed to db/db medium (Fig 3A) could not be fit by an Akt model in which Thr308 Akt phosphorylation is necessary for phosphorylation at Ser473. The model-predicted profile of 2-DG uptake in the presence of db/db medium (Fig 3D) was computed here by reducing the rate constants that regulate GLUT4 translocation. Actually, in T2D subjects, GLUT4 mRNA and protein levels are reduced in adipose tissue (GLUT4 concentration reduced to 50% of control in [20]) but not in skeletal muscle [5], so a defective regulation of GLUT4 translocation may contribute to insulin resistance in L6 cells exposed to db/db medium. Further, AS160 is inhibited by pAkt(Ser473) and fully activated Akt in the model proposed in [20], whereas AS160 is here inhibited by pAkt(Thr308) and fully activated Akt. Indeed, muscle-specific deletion of rictor in mice reduced pAkt(Ser473) to less than 10% of control, but pAS160(Thr642) was still at 80% of control, with data from glucose and insulin tolerance tests not substantially different from control [50].

### Akt, mTOR and cell proliferation

**Fig 6:**
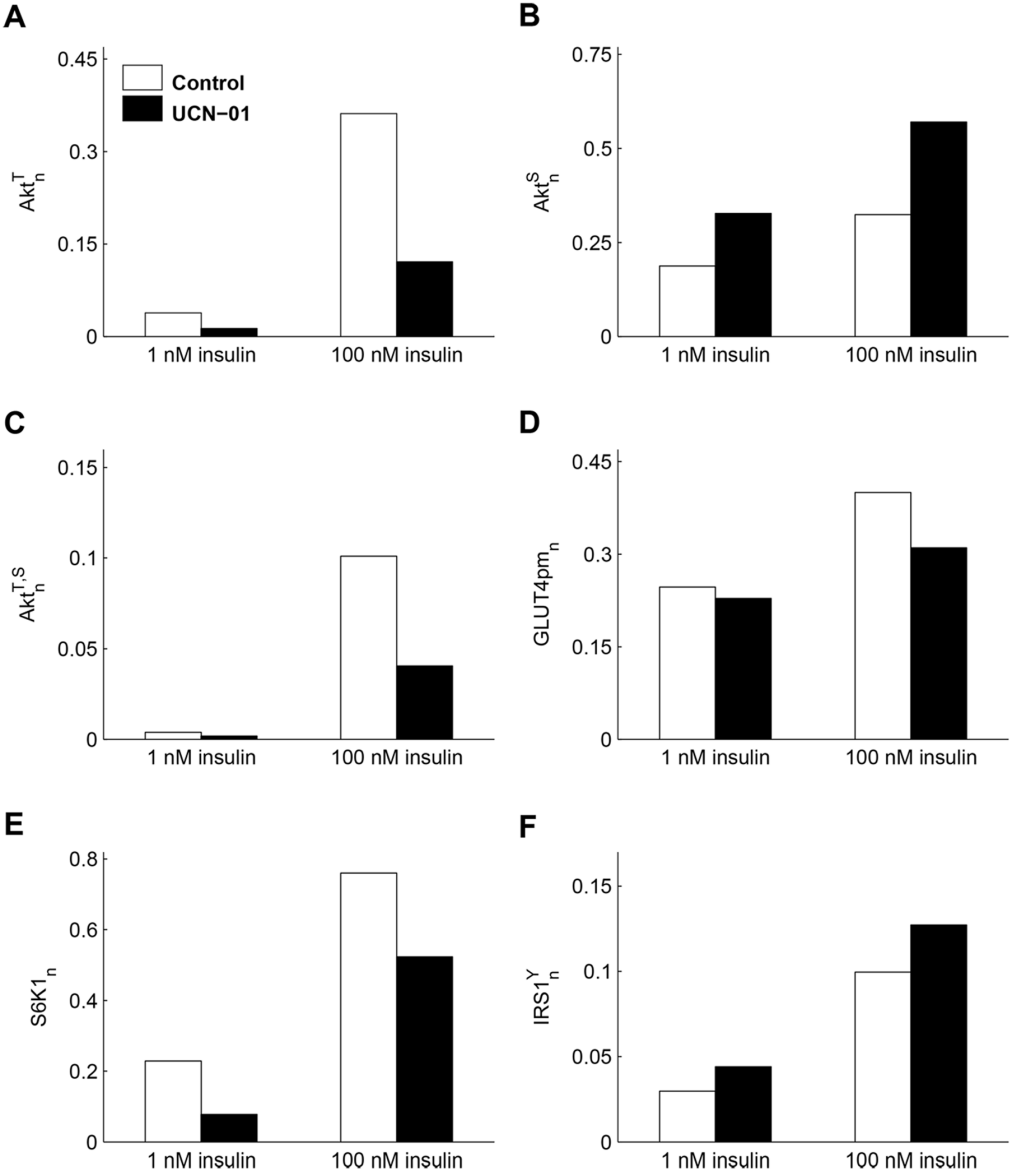
Response of the insulin signaling network to the Akt inhibitor UCN-01 in L6 cells. **(A- C)** Model predictions of $Akt_{n}^{T},\;Akt_{n}^{S}$, and $Akt_{n}^{T,S}$ at 1 and 100 nM insulin in control (white boxes) and in cells exposed to UCN-01 (black boxes), obtained by a tenfold decrease of the PDK1 parameter *a*_9_. The plots show the marked decrease of $Akt_{n}^{T}$ and $Akt_{n}^{T,S}$, with the resulting increase in $Akt_{n}^{S}$ in treated cells compared to control. **(D)** The model prediction of GLUT4 concentration at plasma membrane highlights the insulin resistance elicited by the drug. **(E)** *S*6*K*1_*n*_ reduction due to drug action on PDK1. **(F) $IRS1_{n}^{Y}$** enhancement caused by the weakening of negative feedback.

Since our ISN model focuses on Akt and its phosphorylated forms, we used the model to predict the disjoint effect of the two phosphorylated forms of Akt in antitumor drugs. The protein kinase inhibitor 7-Hydroxystaurosporine (UCN-01) used in cancer therapy induces insulin resistance in humans [51]. UCN-01 inhibits Thr308 but not Ser473 Akt phosphorylation and impairs Akt kinase activity with subsequent inhibition of GLUT4 translocation to cellular membrane [51]. Our model is appropriate to study this effect and Fig 6 shows indeed the marked decrease of $Akt_{n}^{T}$ and $Akt_{n}^{T,S}$, with the resulting insulin resistance elicited by the drug (GLUT4_pm_ increase of 61.8% in control and 36.7% in treated cells, a reduction in the range of glucose transport inhibition reported in [51]).

Fig 5 (panels D-F) and S5 Fig (panel A) highlight the simple relationships found between model-predicted changes in the concentrations of proteins of the ISN, induced by mTOR inhibition, and changes in the AML cell population model parameters that correspond to alterations of the proliferative capacity of the population. The relations found are a rough representation of the complex machinery that regulates cell cycle progression, cell quiescence, and occurrence of cell death. Indeed, we found relationships between the rate constant *λ*_1_ of the G1S transition and different ISN proteins (pAkt(Ser473), pGSK3β(Ser9), FoxO1 and pS6K1(Thr389)), but the molecular pathways that coordinate the action of these proteins in cell cycle regulation remain undetermined. Similarly, pS6K1(Thr389) is related to parameters of cell cycle progression and cell death, but its specific role in these pathways is not specified. The population model of Fig 5A is far from the complexity and richness of behaviors exhibited by the real system. Sophisticated models have been proposed to represent the reactions involving cyclins and cyclin-dependent kinases, see for instance a complex model of cell cycle control in mammalian cells [52].

In summary, the present ISN model permits to investigate the insulin signaling network in the insulin resistance states and in cancer, focusing on the role played by Akt phosphorylated at Ser473 and by the mTOR complexes, as well as on the drug effects. Although the numerical values of model parameters will certainly change with the cell type, the general structure of the model should be considered valid for any cell type as shown by the qualitative agreement observed between model predictions and the experimental data from cell types different from skeletal muscle, such as AML cells, MEFs and HT-29 cells. Model behavior has been tested on a variety of conditions: muscle cells with Pten KO or with induced insulin resistance, knockdown and overexpression of PRAS, TSC2-null cells, cells treated with rapamycin and anticancer drugs such as AZD8055 and UCN-01. The proposed model should thus be useful to integrate the experimental research leading to translationally relevant findings.

## Models

### ISN model equations in normalized form

To reduce the number of unknown parameters, the model equations reported in S1 File (Text S2) are rewritten in a simple non-dimensional form. We assume that synthesis rate and degradation rate constants do not change with time and do not depend on the insulin signal. This assumption may fail during chronic insulin treatment: e.g., after 12 h treatment, IRS1 amount is reduced to 13.6% of control level in 3T3-L1 adipocytes [53]. However, experimental data and model simulations of insulin signaling pathway [10,16,19,20], show that the response to a step insulin increase may reach a steady state in a shorter time, so the horizon is here limited to times that do not include these long-range changes.

The concentrations of all molecules, except insulin and factor J, are normalized to the ratio of production rate *b* (expressed as concentration·time^−1^) over degradation rate constant *μ* (time^−1^) and denoted by the subscript *n*. All normalized concentrations are nonnegative and smaller than the unity for any value of total insulin concentration *I*_*e*_. The model parameters are modified accordingly, and are combinations of the original parameters of the kinetic equations of S1 File (Text S2), as reported in S1 Table.

From Eqs (25)-(26) in S1 File (Text S2) we obtain the equation for total tyrosine phosphorylated IR in normalized form (i.e., normalized to *b*_*IR*_/*μ*_*IR*_). Assuming for simplicity that the positive feedback is not active, we set *PTP*1*B*_*n*_ = 1 and we have the following simplified expression:

$$IR_{n}^{Y}=\frac{IR^{Y}+IR_{b}^{Y}}{\left(b_{IR}/\mu_{IR}\right)}=\frac{a_{b}+I}{a_{b}+a_{0}+I},$$ (1)

where ${IR}_{b}^{Y}\;=\;a_{b}/(a_{b}+a_{0})$ represents the basal (no insulin) tyrosine autophosphorylation, and the free insulin concentration *I* is expressed as a function of total insulin *I*_*e*_ by

$$I=\frac{1}{2}\left(-\left(a_{b}+a_{0}+a_{1}-I_{e}\right)+\sqrt{{\left(a_{b}+a_{0}+a_{1}-I_{e}\right)}^{2}+4\left(a_{b}+a_{0}\right)I_{e}}\right).$$ (2)

Since ${IR}_{b}^{Y}$ can be experimentally measured, *a*_*b*_ can be written as *a*_0_ *ρ*/(1−*ρ*), where ρ denotes this experimental value. Instead of *a*_0_ and *a*_1_, we used the two following quantities that have a more evident meaning: the value *I*_*e*,0.5_ of *I*_*e*_ at ${IR}_{n}^{Y}\;=\;0.5$, and the slope of ${IR}_{n}^{Y}$ at *I*_*e*,0.5_, *S*_0.5_. From Eqs (1) and (2) these quantities are obtained as

$$I_{e,0.5}=\left(a_{0}+\frac{a_{1}}{2}\right)\frac{1-2\rho }{1-\rho }\;,\;\;\;\;\;\;S_{0.5}=\frac{1-\rho }{a_{1}+4a_{0}\left(1-\rho \right)},$$

where *I*_*e*,0.5_ has the dimension of a concentration and *S*_0.5_ is a concentration^–1^.

If the protein phosphatase *PP* is constant, Eqs (33)-(34) in S1 File (Text S2) are rewritten with *IRS*1^*Y*^ and *IRS*1^*S*^ normalized to *b*_*IRS*1_/*μ*_*IRS*1_ as follows:

$$IRS1_{n}^{Y}=\frac{IR_{n}^{Y}}{\left(a_{2}+a_{3}PTP1B_{n}\right)\left(1+a_{4}S6K1_{n}\right)+IR_{n}^{Y}}$$ (3)

$$IRS1_{n}^{S}=\frac{\left(a_{2}+a_{3}PTP1B_{n}\right)a_{4}S6K1_{n}}{\left(a_{2}+a_{3}PTP1B_{n}\right)\left(1+a_{4}S6K1_{n}\right)+IR_{n}^{Y}},$$

where all the parameters are non-dimensional. Eq (35) of S1 File (Text S2) becomes

$$PTP1B_{n}=\frac{1}{1+a_{P}Akt_{n}^{S,T}}.$$ (4)

In the absence of the positive feedback, *a*_*P*_ = 0 and *PTP*1*B*_*n*_ = 1. From Eq (3) it is found, as expected, that $IRS1_{n}^{Y}$ increases with $IR_{n}^{Y}$ and so with *I*_*e*_, provided that *PTP*1*B*_*n*_ and *S*6*K*1*n* are constant. However, the downstream kinase S6K1 exerts an inhibitory effect on the IRS1 tyrosine phosphorylation (negative feedback). In addition, the fully phosphorylated Akt inhibits the phosphatase PTP1B as seen by Eq (4), so Akt phosphorylation might exert a positive feedback on insulin signaling, especially upon inhibition of S6K1.

Proceeding similarly, Eq (36) of S1 File (Text S2) normalized to *b*_*PI*3*K*_/*μ*_*PI*3*K*_ rewrites as

$$PI3K_{n}=\frac{a_{6}{\left(IRS1_{n}^{Y}\right)}^{2}}{1+a_{6}^{\prime }IRS1_{n}^{Y}+a_{6}{\left(IRS1_{n}^{Y}\right)}^{2}},$$ (5)

and (37)-(38) become

$$PIP_{3n}=\;\frac{PI3K_{n}}{a_{7}+a_{8}PTEN_{n}+PI3K_{n}}$$ (6)

$$PDK1_{n}=\frac{a_{9}PIP3_{n}}{1+a_{9}PIP3_{n}}.$$ (7)

For the mTOR complex 2, Eq (39) of S1 File (Text S2) provides the following equation:

$$mTORC2_{n}=\frac{a_{10}PIP_{3n}+a_{11}\;J}{1+\left(1+a_{12}S6K1_{n}\right)\left(a_{10}^{\mu }+a_{10}PIP_{3n}+a_{11}\;J\right)}$$ (8)

where *J* is the factor that induces insulin resistance possibly via the IGF1 receptor [11], and *a*_11_ is the inverse of a concentration.

Since Thr308 and Ser473 Akt phosphorylation may be independently activated [8] two pathways may be followed to achieve full Akt activation, as shown in Fig 1. Assuming the phosphatases *PP*2*A* and *PHLPP* (and so *γ*, *δ*, *ε*, *η*, *θ*, see S1 File (Text S2) constant, Eqs (40)–(42) for the phosphorylated Akt are rewritten as follows:

$$Akt_{n}^{T}=\frac{PDK1_{n}}{D_{1}^{\prime }}\left(a_{13}^{\delta }a_{17}^{\gamma }a_{\theta \varepsilon }\;PDK1_{n}+a_{15}^{\varepsilon }\left(a_{17}^{\delta }-a_{17}^{\gamma }a_{\eta \delta }\right)mTORC2_{n}+a_{13}^{\delta }\right)$$ (9)

$$Akt_{n}^{S}=\frac{mTORC2_{n}}{D_{1}^{\prime }}\left(a_{15}^{\varepsilon }a_{19}^{\gamma }a_{\eta \delta }\;mTORC2_{n}+a_{13}^{\delta }\left(a_{19}^{\varepsilon }-a_{19}^{\gamma }a_{\theta \varepsilon }\right)PDK1_{n}+a_{15}^{\varepsilon }\right)$$ (10)

$$Akt_{n}^{S,T}=\frac{PDK1_{n}mTORC2_{n}}{D_{1}^{\prime }}\left(a_{15}^{\varepsilon }a_{17}^{\delta }a_{19}^{\gamma }mTORC2_{n}+a_{13}^{\delta }a_{19}^{\varepsilon }a_{17}^{\gamma }PDK1_{n}+a_{15}^{\varepsilon }a_{17}^{\gamma }\;+a_{13}^{\delta }a_{19}^{\gamma }\right)$$ (11)

with

$$D_{1}^{\prime }=a_{13}^{\delta }a_{17}^{\gamma }PDK1_{n}^{2}\left(a_{19}^{\varepsilon }mTORC2_{n}+a_{\theta \varepsilon }\right)+a_{15}^{\varepsilon }a_{19}^{\gamma }mTORC2_{n}^{2}\left(a_{17}^{\delta }PDK1_{n}+a_{\eta \delta }\right)+PDK1_{n}mTORC2_{n}\left(a_{13}^{\delta }\left(a_{19}^{\varepsilon }-a_{19}^{\gamma }a_{\theta \varepsilon }\right)+a_{15}^{\varepsilon }\left(a_{17}^{\delta }-a_{17}^{\gamma }a_{\eta \delta }\right)+a_{17}^{\gamma }\left(a_{15}^{\varepsilon }+a_{19}^{\varepsilon }a_{\eta \delta }\right)+a_{19}^{\gamma }\left(a_{13}^{\delta }+a_{17}^{\delta }a_{\theta \varepsilon }\right)-a_{17}^{\delta }a_{19}^{\varepsilon }\right)+PDK1_{n}\left(a_{13}^{\delta }+a_{17}^{\gamma }a_{\theta \varepsilon }\right)+mTORC2_{n}\left(a_{15}^{\varepsilon }+a_{19}^{\gamma }a_{\eta \delta }\right)+1.$$

Eqs (9)–(11) show that in the absence of insulin, when the concentrations of PDK1, Akt^T^ and Akt^S,T^ are likely to be small, Akt^S^ concentration may still be large because of factor J signaling via mTORC2. This behavior cannot be described by a hierarchical scheme in which the Thr308 Akt phosphorylation is necessary for the phosphorylation at Ser473. Akt phosphorylation at threonine and serine measured at zero insulin may be related to basal autophosphorylation of insulin receptor.

Concerning the Akt substrates, the normalized cytoplasmic FoxO1 is given by

$$FoxO1_{n}=\frac{a_{21}\left(Akt_{n}^{S}+Akt_{n}^{T,S}\right)}{1+a_{21}\left(Akt_{n}^{S}+Akt_{n}^{T,S}\right)},$$ (12)

and the normalized concentration of phosphorylated GSK3β is

$$GSK3\beta_{n}=\frac{a_{23}\left(Akt_{n}^{T}+Akt_{n}^{T,S}\right)}{1+a_{23}^{\prime }W+a_{23}\left(Akt_{n}^{T}+Akt_{n}^{T,S}\right)},$$ (13)

where *W* represents the putative factor that promotes GSK3β sequestration [54].

Eq (45) becomes

$$mTORC1_{n}=\frac{a_{24}\left(Akt_{n}^{T}+Akt_{n}^{T,S}\right)}{1+a_{24}\left(Akt_{n}^{T}+Akt_{n}^{T,S}\right)}.$$ (14)

For the activation of S6K1, Thr229 S6K1 phosphorylation by PDK1 follows Thr389 phosphorylation by mTORC1 [55]. So from (46) we have:

$$S6K1_{n}=\frac{a_{26}mTORC1_{n}\cdot \;a_{27}PDK1_{n}}{1+a_{26}mTORC1_{n}+\left(a_{26}^{\mu }+a_{26}mTORC1_{n}\right)a_{27}PDK1_{n}}.$$ (15)

Eq (47) for the normalized *GLUT*4_*pm*_(here denoted as *GLUT*4_*n*_) is rewritten as

$$GLUT4_{n}=\frac{a_{28}+a_{29}\left(Akt_{n}^{T}+Akt_{n}^{T,S}\right)}{1+a_{30}\left(Akt_{n}^{T}+Akt_{n}^{T,S}\right)},$$ (16)

where *a*_28_ < 1, *a*_29_, and *a*_30_ (with *a*_29_ < *a*_30_) may be easily derived from the expression of *GLUT*4_*pm*_ in (47), see S1 File (Text S2). Parameters *a*_28_, *a*_29_, *a*_30_ account in a simple way for the various steps that promote GLUT4 translocation to plasma membrane [6].

Given the concentrations of insulin and of the factor J, Eqs (1)–(16) provide the dose-response curve of each component in the ISN scheme of Fig 1. Note that all model parameters, except *I*_*e*,0.5_ and *S*_0.5_, are nondimensional, and are generally a ratio between the specificity constant times the ratio *b*/*μ* of the kinase, and the same quantity of the phosphatase. In some cases, a rate constant substitutes the specificity constant.

#### Parameter estimation

Eqs (1)–(16) were solved numerically by reducing to a system of only two variables, for instance *PDK*1_*n*_ and *mTORC*2_*n*_. The parameters were estimated by fitting model solutions to data through minimization of a least-squares index. We used a local optimization routine implementing a derivative-free algorithm for bound constrained optimization (Package SDBOX available at the Software Library of the Department of Computer and System Science, Sapienza University of Rome) and the parameters were constrained to be nonnegative. Since *a*_0_ and *a*_1_ are related to *I*_*e*,0.5_ and *S*_0.5_ by the equations

$$a_{0}=\left(\frac{1}{2S_{0.5}}-\frac{I_{e,0.5}}{1-2\rho }\right)\frac{1-\rho }{1-2\rho }\;,\;\;\;\;\;\;a_{1}=\left(4I_{e,0.5}\frac{1-\rho }{1-2\rho }-\frac{1}{S_{0.5}}\right)\frac{1-\rho }{1-2\rho },$$

the terms in parenthesis were constrained to be positive to ensure the positivity of *a*_0_ and *a*_1_. Moreover, in view of the meaning of parameters (see S1 File (Text S2)), the quantities $\;a_{17}^{\delta }-a_{17}^{\gamma }a_{\eta \delta }$ and $a_{19}^{\varepsilon }-a_{19}^{\gamma }a_{\theta \varepsilon }$ in Eqs (9)–(11) were constrained to be positive. Also, we considered that $a_{17}^{\gamma }a_{19}^{\varepsilon }a_{\eta \delta }{+\;a}_{19}^{\gamma }a_{17}^{\delta }a_{\theta \varepsilon }-a_{17}^{\delta }a_{19}^{\varepsilon }$ in $D_{1}^{'}$ equals ${a_{17}^{\delta }a_{19}^{\varepsilon }\;(\mu }_{Akt}/\gamma )$ with *μ*_*AKt*_/*γ* ≪ 1.

To further reduce the number of parameters to be estimated, *a*_2_, *a*_7_ and $a_{10}^{\mu }$ in Eqs (3), (6) and (8), that are likely to be small, were set to zero and we assumed *a*_9_ = *a*_10_ (see S2 Table) as no data on the phosphorylation of PDK1 and mTORC2 were available.

In particular, for the parameter estimation of C2C12 data, we assumed that: i) the factor J is negligible and then *a*_11_ in Eq (8) was set to zero; ii) $a_{23}^{'}$ in Eq (13) was also set to zero; iii) the parameter *a*_*P*_ of positive feedback in Eq (4) was set to zero because IR phosphorylation was similar in control and PTEN-silenced cells (see Fig 2 panel A of main text); iv) basal IR autophosphorylation at zero insulin was set to 0.03 in view of the data in [32].

We fit experimental data of L6 cells with the following assumptions: i) J has negligible concentration in control medium and a larger concentration, to be estimated, in db/db medium; ii) insulin resistance also occurs because of increased IRS1 degradation via a negative feedback due to enhancement of mTORC2 signaling [35]. Therefore, to fit the data of cells exposed to db/db medium, we reduced the values of $a_{6}^{'}$ and *a*_6_ in Eq (5) according to a twofold increase of IRS1 degradation rate constant (*μ*_*IRS*1_); iii) we changed $Akt_{n}^{T}$ into $Akt_{n}^{S}$ in Eq (13) of GSK3β and estimated $a_{23}^{'}$ with *W* = 1 for db/db data; iv) the action of Rapamycin was accounted for by reducing *a*_24_ in Eq (14) by a factor 0.1, and the action of PP242 by reducing *a*_10_ and *a*_11_ in Eq (8) and *a*_24_ by a factor 0.15. S2 Table reports the estimates of model parameters obtained from the data of C2C12 and L6 cells.

The sensitivities of the normalized concentrations of proteins with respect to model parameters were computed as derivative of log concentration with respect to log parameter at the optimum. The use of these (relative) sensitivities provides nondimensional quantities that do not depend on the absolute values of concentrations and parameters. Sensitivities were evaluated numerically upon a ±10% perturbation of the parameters. The results are presented in the form of matrices where numerical values, which are positive for a positive regulation and negative for an inhibition, are converted to a color.

### Model of cell population response to mTOR inhibitor with antitumor activity

To analyze the behavior of the AML cell population we used a mathematical model based on the age formalism, represented by the block diagram of Fig 5A. Denoting by *N*_1_(*t*) the number of G0/G1 cells at time *t*, and by *n*_*i*_(*a*_*i*_, *t*) the cell density at time *t* with respect to age *a*_*i*_, with *i* = 2,3 for cells in S and G2M phases, we have the balance equations

$$\frac{d}{dt}N_{1}=-\left(\lambda_{1}+\mu^{\prime }\right)N_{1}\left(t\right)+2n_{3}\left(T_{3},t\right)\;,$$ (17)

$$\frac{\partial }{\partial t}n_{i}\left(a_{i},t\right)+\frac{\partial }{\partial a_{i}}n_{i}\left(a_{i},t\right)=-\mu^{\prime }n_{i}\left(a_{i},t\right)\;,\;\;\;\;\;\;i=2,3,$$ (18)

with boundary conditions

$$n_{2}\left(0,t\right)=\lambda_{1}N_{1}\left(t\right)\;,\;\;\;n_{3}\left(0,t\right)=n_{2}\left(T_{2},t\right),$$

where *λ*_1_ is the rate constant that regulates the G1 to S phase transition, *T*_2_ and *T*_3_ are the transit times in S and, respectively, G2M phases, and *μ*′ is the rate constant (taken equal for all cycle phases) of cell loss from the compartments of viable cells. The number of S-phase cells at time *t*, *N*_2_(*t*), is given by $\int_{0}^{T_{2}}n_{2}(a_{2},t)da_{2}$, and similarly for the number of G2M cells, *N*_3_(*t*).

We observe that, in this simple scheme of cell progression across cell cycle, the rate constant *λ*_1_ represents the activity of the cyclins (as cyclin D) and the cyclin-dependent kinases that regulate the G1 to S transition. The rate constant *μ*′ may be related to the activity of the proteins that regulate the autophagia and/or the early phases of apoptosis.

As in [45], we assumed that cells lost from the viable population can still be transiently measurable, and so are counted in the phase fractions obtained by flow cytometry (see Fig 3 in Ref [44]). Denoting the number of cells lost from the three viable compartments by *D*_*i*_(*t*), *i* = 1–3 (Fig 5A in the main text), we write

$$\frac{d}{dt}D_{i}=-\mu^{\prime \prime }D_{i}\left(t\right)+\mu^{\prime }N_{i},$$ (19)

with *μ*′′ a rate constant of cell fragmentation related to apoptosis. Apoptotic bodies and fragments are eventually gathered in a further compartment that obeys the equation

$$\frac{d}{dt}A=-\mu_{deg}A\left(t\right)+\mu^{\prime \prime }\left(D_{1}\left(t\right)+D_{2}\left(t\right)+D_{3}\left(t\right)\right),$$ (20)

where the material that leaves this compartment with rate constant *μ*_*deg*_ is no longer measurable.

Assuming that the cell population is in balanced exponential growth (or is declining under the treatment) with rate constant *α*, the following relation holds:

$$\alpha +\mu^{\prime }+\lambda_{1}=2\lambda_{1}\;e^{-\left(\alpha +\mu^{\prime }\right)\left(T_{2}+T_{3}\right)},$$ (21)

where +*μ*′ > 0. The fractions of cells in the cell cycle phases, measured by PI staining and flow cytometry, are derived accounting for the dead cells in compartments *D*_*i*_(*t*), *i* = 1–3, together with the viable cells in the respective phase. So, for instance, *f*_*G*1_ is computed as (*N*_1_+*D*_1_)/*N*_*tot*_, with *N*_*tot*_ the total amount of cells and fragments. We have:

$$f_{G1}=\frac{\left(\alpha +\mu^{\prime }\right)\left(\alpha +\mu_{deg}\right)\left(\alpha +\mu^{\prime \prime }+\mu^{\prime }\right)}{\left(\alpha +\mu^{\prime }+\lambda_{1}\left(1-e^{-\left(\alpha +\mu^{\prime }\right)\left(T_{2}+T_{3}\right)}\right)\right)\left(\mu^{\prime \prime }\mu^{\prime }+\left(\alpha +\mu_{deg}\right)\left(\alpha +\mu^{\prime \prime }+\mu^{\prime }\right)\right)},$$ (22)

$$f_{S}=\frac{\lambda_{1}\left(1-e^{-\left(\alpha +\mu^{\prime }\right)T_{2}}\right)\left(\alpha +\mu_{deg}\right)\left(\alpha +\mu^{\prime \prime }+\mu^{\prime }\right)}{\left(\alpha +\mu^{\prime }+\lambda_{1}\left(1-e^{-\left(\alpha +\mu^{\prime }\right)\left(T_{2}+T_{3}\right)}\right)\right)\left(\mu^{\prime \prime }\mu^{\prime }+\left(\alpha +\mu_{deg}\right)\left(\alpha +\mu^{\prime \prime }+\mu^{\prime }\right)\right)},$$ (23)

$$f_{G2M}=\frac{\lambda_{1}e^{-\left(\alpha +\mu^{\prime }\right)T_{2}}\left(1-e^{-\left(\alpha +\mu^{\prime }\right)T_{3}}\right)\left(\alpha +\mu_{deg}\right)\left(\alpha +\mu^{\prime \prime }+\mu^{\prime }\right)}{\left(\alpha +\mu^{\prime }+\lambda_{1}\left(1-e^{-\left(\alpha +\mu^{\prime }\right)\left(T_{2}+T_{3}\right)}\right)\right)\left(\mu^{\prime \prime }\mu^{\prime }+\left(\alpha +\mu_{deg}\right)\left(\alpha +\mu^{\prime \prime }+\mu^{\prime }\right)\right)},$$ (24)

and the fraction of apoptotic materials in the subG1 region of the PI fluorescence histogram is given by *f*_*A*_ = 1−*fG*_1_−*f*_*S*_−*f*_*G*2*M*_. The total fraction of dead cells and fragments is

$$f_{Dead}=\frac{\mu^{\prime }\left(\alpha +\mu_{deg}+\mu^{\prime }\right)}{\mu^{\prime \prime }\mu^{\prime }+\left(\alpha +\mu_{deg}\right)\left(\alpha +\mu^{\prime \prime }+\mu^{\prime }\right)}.$$ (25)

The effect of the drug on cell cycle progression, assessed by (^3^H)thymidine incorporation (see S3 Fig in [44]), is measured by the labeling index (LI), which is derived in the above model by writing an equation for the number of labeled cells [45]. We have

$$\text{LI}\left(\Delta \right)=\frac{\lambda_{1}\left(\alpha +\mu^{\prime \prime }\right)}{\left(\alpha +\mu^{\prime }\right)\left(\alpha +\mu^{\prime \prime }+\mu^{\prime }\right)}f_{G1}\left[e^{\alpha \Delta }\left(1-e^{-\left(\alpha +\mu^{\prime }\right)\left(T_{2}+\Delta \right)}\right)+\mu^{\prime }e^{-\mu^{\prime \prime }\Delta }\left(\frac{\left(e^{\left(\alpha +\mu^{\prime \prime }\right)\Delta }-1\right)}{\alpha +\mu^{\prime \prime }}-\frac{e^{-\left(\alpha +\mu^{\prime }\right)T_{2}}\left(e^{\left(\mu^{\prime }-\mu^{\prime \prime }\right)\Delta }-1\right)}{\mu^{\prime }-\mu^{\prime \prime }}\right)\right],$$ (26)

where Δ is the length of the labeling period (Δ = 6 hrs [44]).

#### Parameter estimation

The unknown parameters in Eqs (21)–(26), to be estimated from the available data in the control and in the treated cell populations, are *α*, *λ*_1_, *T*_2_, *T*_3_, *μ*′, *μ*′′ and *μ*_*deg*_. However, Eq (21) is an independent relationship among parameters that actually reduces the number of unknowns. While *α*, *λ*_1_, *T*_2_, *T*_3_, *μ*′, *μ*′′ are expected to be different in the control and in the populations treated with different drug doses, it is likely that *μ*_*deg*_ does not change and, for simplicity, is taken equal to the value of *μ*′′ in the control.

The growth rate constant *α* of the untreated population was estimated from the growth curve of tumor size in the xenograft, and we assumed that the same *α* value holds for the MV4-11 cell line. From the cell fractions in cell-cycle phases and the fraction of PI-positive cells represented by *f*_*Dead*_ in Eq (25), we estimated *λ*_1_, *T*_2_, *T*_3_, *μ*′, *μ*′′ by least squares using Eqs (21)–(25). Eq (26) provided the value of the labeling index in control, not given in [44]. The value of *α* in treated cells was not available. The LI values in treated populations, reported in S3 Fig of [44] as ratios treated/control, were multiplied by the LI of control (31.2%) to obtain the actual values to be compared with model-predicted LI.

## Supporting Information

S1 FigExperimental data of C2C12 myoblasts (not used in the parameter estimation) and model predictions.Data (mean ± SEM) in panels A and B are replotted from Ref [32] of main text. **(A)** Relative pAS160 (Thr642) concentration in control (black) and PTEN-suppressed (red) cells, together with the dose-response curves predicted by the model. The equation for pAS160 (Thr642) (inactive form) is given by $pAS160_{n}\;=\;0.5\;(Akt_{n}^{T}+Akt_{n}^{T,S})/[1+0.5\;(Akt_{n}^{T}+Akt_{n}^{T,S})]$. **(B)** Relative PIP3 concentration in control (black squares) and PTEN-suppressed (red squares) cells with model prediction at zero insulin (white boxes) and 10 nM insulin (blue boxes). (**C, D**) Fitting of the relative pAkt(Ser473) and prediction of relative PIP3 in the hypothesis that mTORC2 is activated by PI3K instead of PIP3. **(E)** Model prediction of *PDK*1*_n_* in control and PTEN-suppressed cells at zero (white boxes) and 100 nM insulin (gray boxes). **(F)** Model prediction of total *pAkt_n_* in control and PTEN-suppressed cells at zero (white boxes) and 100 nM insulin (gray boxes).(PDF)

S2 Fig Sensitivity analysis for the ISN model of C2C12 myocytes. The plot shows the sensitivities of protein concentrations to the estimated parameters of the model at the extracellular insulin concentration of 44.68 nM.
(PDF)

S3 FigModel predictions for L6 myotubes.**(A, B)** Model prediction of $IRS1_{n}^{Y}$ (panel A) and $IRS1_{n}^{S}$ (panel B) for cells in control medium at zero and 10 nM insulin in the absence of inhibitor (green), and in the presence of 50 nM rapamycin (yellow) and 500 nM PP242 (pink). **(C, D)** Model predictions as in (A) and (B), but for cells exposed to db/db medium. Panels A-D show the different effect of decreased negative feedback on tyrosine and serine residues of IRS1. **(E)** Plot of values assumed by *PDK*1*_n_* (abscissa) and *mTORC*2*_n_* (ordinate) when *I* *_e_* increases from zero to 100 nM for control and db/db medium. **(F)** 3D plot of $kt_{n}^{T},\;Akt_{n}^{S}$, and $Akt_{n}^{T,S}$ as a function of *PDK*1*_n_* and *mTORC*2*_n_* according to Eqs (9)–(11) in Main Text. With the present estimates of Akt model parameters, $Akt_{n}^{S}$ increases with *mTORC*2*_n_* and decreases with *PDK*1*_n_*, while $Akt_{n}^{T,S}$, and less clearly $Akt_{n}^{T}$, increase with both *PDK*1*_n_* and *mTORC*2*_n_*.(PDF)

S4 Fig Sensitivity analysis for the ISN model of L6 myotubes. The plot shows the sensitivities of protein concentrations to the estimated parameters of the model at the extracellular insulin concentration of 9.69 nM for control cells (upper panel) and cells in db/db medium (lower panel).
(PDF)

S5 Fig ISN response to the mTOR inhibitor AZD8055. (**A**) Relationship between the decrease of pS6K1 (Thr389) (squares) and that of *λ*_1_ at increasing concentrations of the drug. The fitting line has equation *y* = 1.10 *x*/(0.35·10^-2^+*x*), with y = pS6K1 (Thr389) and *x* = *λ*_1_. A similar function fits the relation between FoxO1_cyt_ (triangles) and *λ*_1_. Data are normalized to control and represented for the different drug concentrations as control (blue), AZD10 (red), AZD100 (green), AZD1000 (cyan). (**B**) Normalized data (mean ± SD) of pS6K1 (Thr389) vs AZD8055 concentration (green diamonds) replotted from [44] and model outputs (black squares), together with the fitting line *y* = 6.35/(6.34+*x*^0.716^). **(C, D)** Decrease of pAkt (Ser473) (panel C) and of the ratio between cytosolic and nuclear FoxO1 concentration (panel D) with increasing AZD8055 concentration as computed by the present model (black squares). Fitting lines have equations similar to that in panel B. The green diamonds and lines, replotted from Ref [43], show the decrease of the same proteins in different cells.
(PDF)

S1 File Supporting Information Text. Text S1: Summary of reactions within the insulin signaling network. Text S2: Kinetic and equilibrium equations. Text S3: Improved activation model of Akt and mTOR complexes.
(DOCX)

S1 Table Expression of ISN model parameters in Eqs (1)–(16) of section Models in terms of the kinetic parameters of the equations in S1 File (Text S2). (PDF)

S2 Table Parameters in Eqs (1)–(16) in Models estimated from data of C2C12 and L6 cells. (PDF)

S3 Table Estimates of parameters of the cell population model in control and in cells exposed to 10, 100, and 1000 nM AZD8055. (PDF)
