# Supplementary figures and images for: Insulin Signaling in Insulin Resistance States and Cancer: A Modeling Analysis
Source: PLoS One. 2016 May 5;11(5):e0154415. doi: 10.1371/journal.pone.0154415 (PMC4858213; doi:10.1371/journal.pone.0154415)

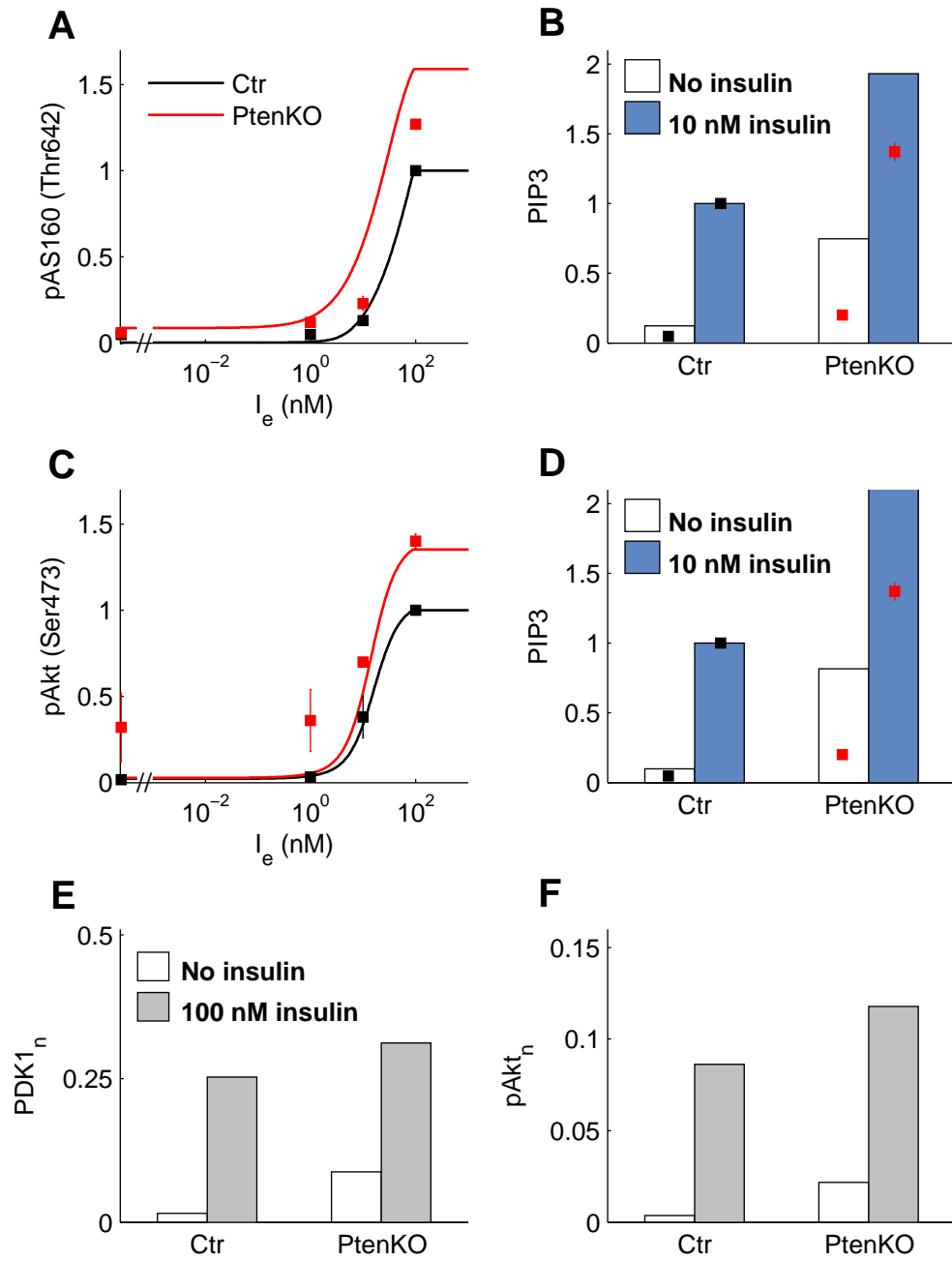

Supplement: S1 Fig — Data (mean ± SEM) in panels A and B are replotted from Ref [32] of main text. (A) Relative pAS160 (Thr642) concentration in control (black) and PTEN-suppressed (red) cells, together with the dose-response curves predicted by the model. The equation for pAS160 (Thr642) (inactive form) is given by pAS160n = 0.5 (AktnT+AktnT,S)/[1+0.5 (AktnT+AktnT,S)]. (B) Relative PIP3 concentration in control (black squares) and PTEN-suppressed (red squares) cells with model prediction at zero insulin (white boxes) and 10 nM insulin (blue boxes). (C, D) Fitting of the relative pAkt(Ser473) and prediction of relative PIP3 in the hypothesis that mTORC2 is activated by PI3K instead of PIP3. (E) Model prediction of PDK1n in control and PTEN-suppressed cells at zero (white boxes) and 100 nM insulin (gray boxes). (F) Model prediction of total pAktn in control and PTEN-suppressed cells at zero (white boxes) and 100 nM insulin (gray boxes). (PDF) [file pone.0154415.s001.pdf]

Sensitivity Analysis C2C12

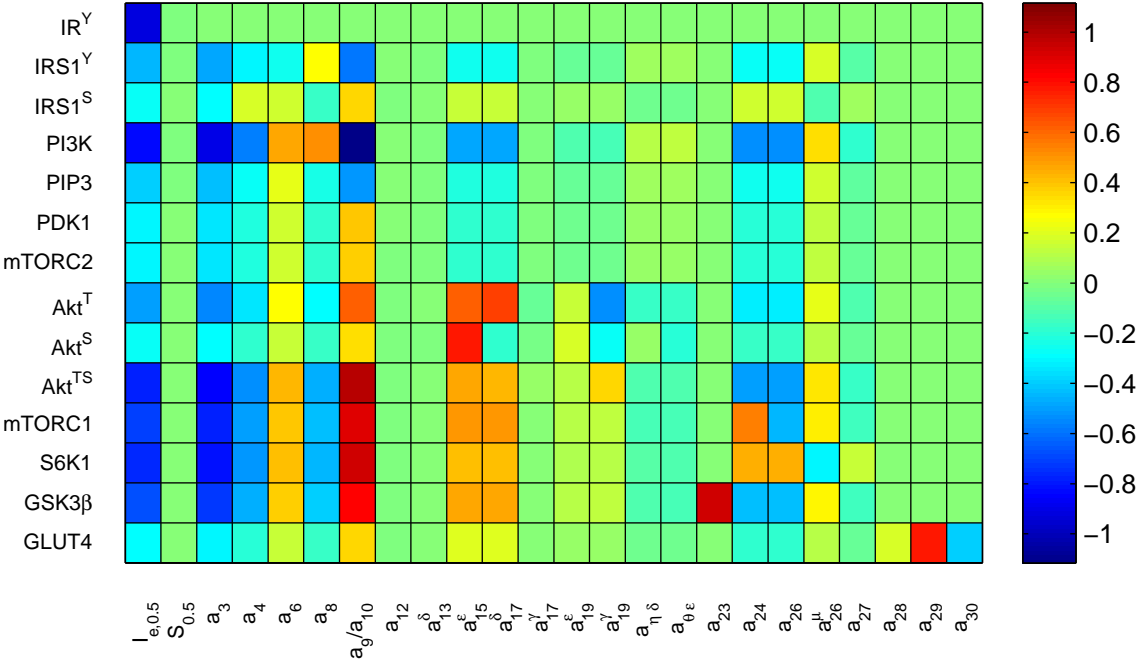

Supplement: S2 Fig — The plot shows the sensitivities of protein concentrations to the estimated parameters of the model at the extracellular insulin concentration of 44.68 nM. (PDF) [file pone.0154415.s002.pdf]

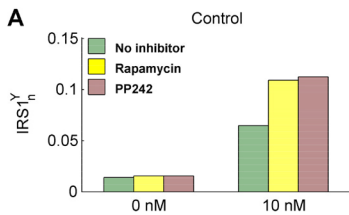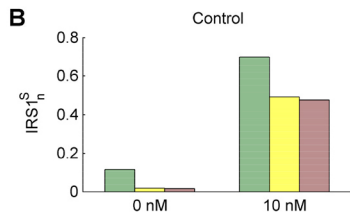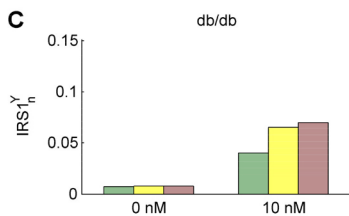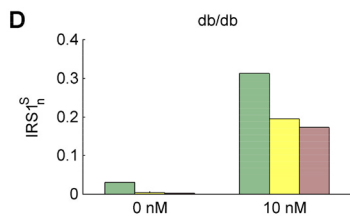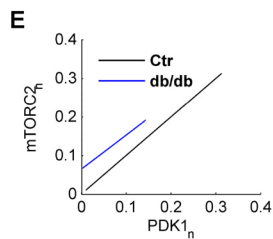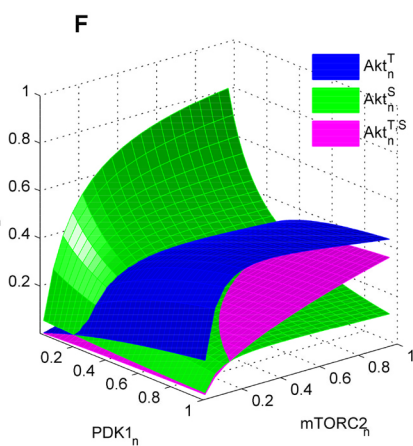

Supplement: S3 Fig — (A, B) Model prediction of IRS1nY (panel A) and IRS1nS (panel B) for cells in control medium at zero and 10 nM insulin in the absence of inhibitor (green), and in the presence of 50 nM rapamycin (yellow) and 500 nM PP242 (pink). (C, D) Model predictions as in (A) and (B), but for cells exposed to db/db medium. Panels A-D show the different effect of decreased negative feedback on tyrosine and serine residues of IRS1. (E) Plot of values assumed by PDK1n (abscissa) and mTORC2n (ordinate) when I e increases from zero to 100 nM for control and db/db medium. (F) 3D plot of ktnT, AktnS, and AktnT,S as a function of PDK1n and mTORC2n according to Eqs (9)–(11) in Main Text. With the present estimates of Akt model parameters, AktnS increases with mTORC2n and decreases with PDK1n, while AktnT,S, and less clearly AktnT, increase with both PDK1n and mTORC2n. (PDF) [file pone.0154415.s003.pdf]

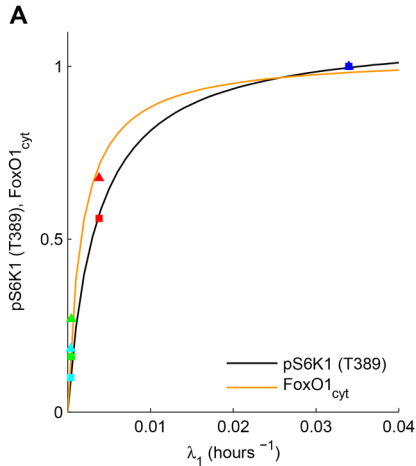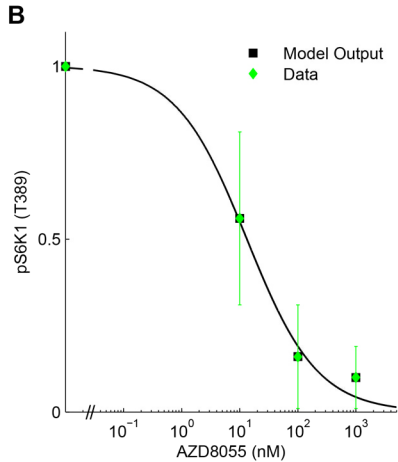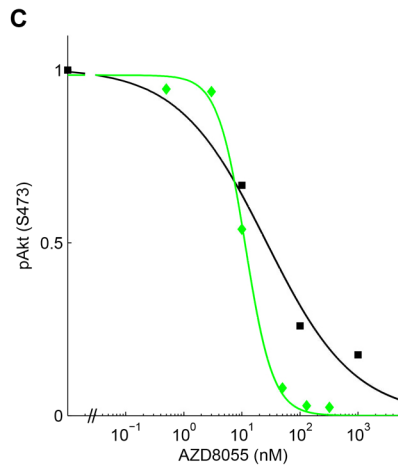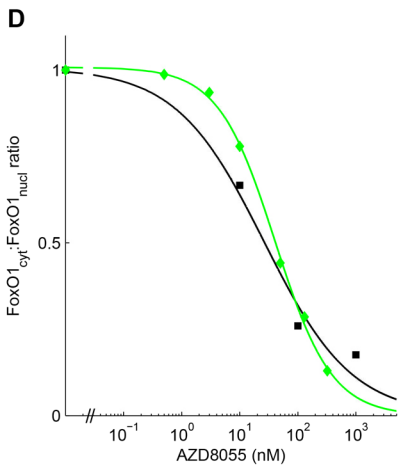

Supplement: S5 Fig — (A) Relationship between the decrease of pS6K1 (Thr389) (squares) and that of λ1 at increasing concentrations of the drug. The fitting line has equation y = 1.10 x/(0.35·10-2+x), with y = pS6K1 (Thr389) and x = λ1. A similar function fits the relation between FoxO1cyt (triangles) and λ1. Data are normalized to control and represented for the different drug concentrations as control (blue), AZD10 (red), AZD100 (green), AZD1000 (cyan). (B) Normalized data (mean ± SD) of pS6K1 (Thr389) vs AZD8055 concentration (green diamonds) replotted from [44] and model outputs (black squares), together with the fitting line y = 6.35/(6.34+x0.716). (C, D) Decrease of pAkt (Ser473) (panel C) and of the ratio between cytosolic and nuclear FoxO1 concentration (panel D) with increasing AZD8055 concentration as computed by the present model (black squares). Fitting lines have equations similar to that in panel B. The green diamonds and lines, replotted from Ref [43], show the decrease of the same proteins in different cells. (PDF) [file pone.0154415.s005.pdf]
