# Supplementary material for: Insulin Signaling in Insulin Resistance States and Cancer: A Modeling Analysis
Source: PLoS One. 2016 May 5;11(5):e0154415. doi: 10.1371/journal.pone.0154415 (PMC4858213; doi:10.1371/journal.pone.0154415)
Supplement: S1 File — Text S1: Summary of reactions within the insulin signaling network. Text S2: Kinetic and equilibrium equations. Text S3: Improved activation model of Akt and mTOR complexes. (DOCX) [file pone.0154415.s006.docx]

### Supplementary Information

### Text S1. Summary of reactions within the insulin signaling network.

### Text S2. Kinetic and equilibrium equations.

Text S3. Improved activation model of Akt and mTOR complexes.

S1 Fig. Experimental data of C2C12 myoblasts (not used in the parameter estimation) and model predictions.

S2 Fig. Sensitivity analysis for the model of C2C12 cells.

S3 Fig. Model predictions for L6 myotubes.

S4 Fig. Sensitivity analysis for the model of L6 cells.

S5 Fig. Response of the insulin signaling network to the mTOR inhibitor AZD8055.

S1 Table. Expression of the ISN model parameters in Eqs. (1)-(16) of section Models in terms of the kinetic parameters of equations in Text S2.

S2 Table. Parameters of the ISN model in Eqs. (1)-(16) of the section Models estimated from data of C2C12 and L6 cells.

S3 Table. Estimates of the parameters of the cell population model in control and in cells exposed to 10, 100, and 1000 nM AZD8055

### S1 Text. Summary of reactions within the insulin signaling network

The reactions listed and discussed here follow the scheme of the IR-PI3K-Akt-mTOR network considered in the present study and illustrated in Fig 1.

#### **(I) IR phosphorylation/dephosphorylation and inhibition of dephosphorylation by pAkt**

We neglect the binding of two insulin molecules to IR, as well as receptor internalization and recycling, and treat phosphorylation at multiple tyrosine residues as one-site phosphorylation. Insulin binding and receptor autophosphorylation are represented according to the reaction scheme (phosphate group omitted)

I + IR ⮀ C_0_ → IR^Y^, (1)

where I denotes free insulin, IR the free insulin receptor and C_0_ the complex I:IR. IR^Y^ denotes the tyrosine-phosphorylated, but still insulin-bound, insulin receptor. The reaction constants are *k*_0_*_a_*_,_ *k*_0_*_d_*, *k*_0_*_c_*, and *K_m,_*_0_ = (*k*_0_*_d_* + *k*_0_*_c_*)/*k*_0_*_a_*. Spontaneous IR autophosphorylation at zero insulin, that leads to $IR_{b}^{Y}$, is regulated by a phosphorylation rate constant, *k_b_* .

As shown in Fig 1, IR is dephosphorylated by the protein tyrosine phosphatase 1B (PTP1B) [1]. Akt may inactivate PTP1B upon phosphorylation at Ser50, which enhances insulin signaling via a positive feedback loop [2]. IR dephosphorylation and the possible inactivation of PTP1B by the phosphorylated Akt (pAkt) are represented by the reactions

IR^Y^ + PTP1B ⮀ C_1_ → I + IR + PTP1B (2)

pAkt + PTP1B ⮀ C_P_ → PTP1B_i_ + pAkt , (3)

where C_1_ is the complex IR^Y^:PTP1B with *k_1a_* , *k_1d_* and *k_1c_*  the reaction constants and *K_m,_*_1_ = (*k*_1_*_d_* + *k*_1_*_c_*)/*k*_1_*_a_* . The same reaction constants also regulate the complex $IR_{b}^{Y}$:PTP1B, denoted by $C_{1}^{'}$. C_P_ is the complex of PTP1B with the inhibitor pAkt and PTP1B_i_ is the inactive form of PTP1B. The reaction constants are *k_Pa_* , *k_Pd_* , *k_Pc_* , and *K_m_*_,_*_P_* = (*k_Pd_* + *k_Pc_*)/*k_Pa_*. The inverse transition PTP1B_i_ → PTP1B is regulated by a rate constant *k_−P_* . Similar symbols are used in the following.

**(II) *IRS1 phosphorylation/dephosphorylation with serine phosphorylation by S6K1***

For the phosphorylation/dephosphorylation of IRS1 we consider two coupled cycles that account for IRS1 phosphorylation at tyrosine and serine residues [1]. Several IRS1 serine residues are phosphorylated by downstream kinases: e.g., at Ser318 by the atypical protein kinase C-ζ, at Ser632 by mTORC1/S6K1, at Ser307 by mTORC1, at Ser332 by GSK3 [3,4]. Ser302 phosphorylation by S6K1 disrupts IRS1 ability to interact with activated IR [5]. For simplicity, we assume that IRS1 is only phosphorylated by activated S6K1, whereas IR^Y^ (that denotes here spontaneous plus insulin-induced tyrosine phosphorylation) phosphorylates IRS1 at tyrosine residues. We have:

IRS1 + IR^Y^ ⮀ C_2_ → IRS1^Y^ + IR^Y^ (4)

IRS1^Y^ + PTP1B ⮀ C_3_ → IRS1 + PTP1B (5)

IRS1 + S6K1 ⮀ C_4_ → IRS1^S^ + S6K1 (6)

IRS1^S^ + PP ⮀ C_5_ → IRS1 + PP (7)

where IRS1 is the unphosphorylated insulin receptor substrate-1, IRS1^Y^ is the tyrosine phosphorylated IRS1, and PTP1B dephosphorylates IRS1^Y^. PP is a protein serine phosphatase. C_2_, C_3_, C_4_ and C_5_ are the complexes IR^Y^:IRS1, IRS1^Y^:PTP1B, IRS1:S6K1 and IRS1^S^:PP, respectively, with *k_ia_*, *k_id_* and *k_ic_*  the respective reaction constants and *K_m_*_,_*_i_* = (*k_id_* +*k_ic_*)/*k_ia_*, *i* = 2,3,4,5.

IRS1 phosphorylation at serine residues represents a negative feedback that attenuates insulin signaling [6,7,8,9]. The formation of complex C_P_ (reaction (3)), with the inactivation of PTP1B, represents a positive feedback that attenuates the dephosphorylation of IR^Y^ and IRS1^Y^, see (2) and (5), and enhances insulin signaling.

**(III) *PI3K activation/deactivation***

The formation of PI3K occurs through recruitment of the catalytic subunit p110 to plasma membrane (PM) and p110 binding to the regulatory subunit p85. We assume that both steps of the process are regulated by a complex IRS1^Y^:p85, formed upon tyrosine phosphorylation of IRS1:

IRS1^Y^:p85+ p110_cyt_  ⮀ $C_{6}^{'}$ → IRS1^Y^:p85+ p110_pm_ ⮀ C_6_ → IRS1^Y^ + PI3K , (8)

with the kinetic constants of complexes $C_{6}^{'}$ and C_6_ defined accordingly. Transport of p110 from PM to cytosol and PI3K deactivation occur according to rate constants $k_{-6}^{'}$ and *k_−_*_6_. PI3K activation by IRS-independent mechanisms also occurs upon growth factor stimulation [10].

**(IV) *Phosphatidylinositol phosphates and PDK1***

PI3K phosphorylates the phosphatidylinositol 4,5-bisphosphate PI(4,5)P_2_ on the 3´- position to form the second messenger PI(3,4,5)P_3_ (PIP_3_), whereas PIP_3_ dephosphorylation to PI(4,5)P_2_ occurs through the phosphatase and tensin homologue (PTEN) 3´-lipid phosphatase [11]. We neglect PIP_3_ formation from PI(3,4)P_2_ and the SHIP1/2 phosphatases. PIP_3_ activates the phosphoinositide-dependent protein kinase-1 (PDK1) [12]. We have:

PI(4,5)P_2_ + PI3K ⮀ C_7_ → PIP_3_ + PI3K (9)

PIP_3_ + PTEN ⮀ C_8_ → PI(4,5)P_2_ + PTEN (10)

PIP_3_ + PDK1_i_ ⮀ C_9_ → PIP_3_ + PDK1 . (11)

C_7_, C_8_, C_9_ denote the complexes in (9)-(11) and the kinetic constants are defined accordingly. The transition from active to inactive form of PDK1, PDK1_i_ , is regulated by the rate constant *k_−_*_9_.

**(V)  *Activation/deactivation of mTORC2***

It is currently accepted that the mTOR complex 2 is the kinase that promotes Akt phosphorylation at Ser473 [13]. As suggested in [14], mTORC2 resides at the plasma membrane and is regulated by growth factors. PIP_3_ may also activate mTORC2, see reaction (12). The inverse transition to the inactive form, mTORC2 → mTORC2_i_ , is regulated by *k_−_*_10_. In the present model mTORC2 activation may be promoted independently by a factor, denoted as J in reaction (13), which is related to signaling from the proximal small intestine possibly via the IGF1 receptor [15]. The inverse transition is regulated by *k_−_*_11_. We have:

mTORC2_i_ + PIP_3_ ⮀ C_10_ → mTORC2 + PIP_3_ (12)

mTORC2_i_ + J ⮀ C_11_ → mTORC2 + J (13)

where subscript “i” denotes the inactive form and C_10_, C_11_ are the complexes in reactions (12)-(13). Moreover, S6K1 negatively regulates mTORC2 by Rictor phosphorylation at Thr1135, which impairs mTORC2 ability to phosphorylate Akt at Ser473 [16,17]:

mTORC2 + S6K1 ⮀ C_12_ → mTORC2^T^ + S6K1, (14)

where mTORC2^T^ denotes the mTOR complex 2 with Rictor phosphorylated at Thr1135. The inverse transition is regulated by *k_−_*_12_.

**(VI)  *Akt phosphorylation/dephosphorylation***

The first step in Akt activation is the protein translocation to plasma membrane through interaction with PIP_3_. For simplicity, we have not considered this step as well as the recruitment of PDK1 to PM, assuming that Akt translocation to PM and Thr308 Akt phosphorylation are a unique step directly regulated by PDK1. At the membrane, mTORC2 phosphorylates Akt at Ser473. The protein phosphatase PHLPP dephosphorylates Akt at Ser473 and the phosphatase PP2A dephosphorylates Akt at Thr308. As Thr308 and Ser473 phosphorylation may be independently activated [18], two pathways may be followed to achieve full Akt activation. We have:

Akt + PDK1 ⮀ C_13_ → Akt^T^ + PDK1 (15)

Akt^T^ + PP2A ⮀ C_14_ → Akt + PP2A (16)

Akt + mTORC2 ⮀ C_15_ → Akt^S^ + mTORC2 (17)

Akt^S^ + PHLPP ⮀ C_16_ → Akt + PHLPP (18)

Akt^S^ + PDK1 ⮀ C_17_ → Akt^S,T^ + PDK1 (19)

Akt^S,T^ + PP2A ⮀ C_18_ → Akt^S^ + PP2A (20)

Akt^T^ + mTORC2 ⮀ C_19_→ Akt^T,S^ + mTORC2 (21)

Akt^T,S^ + PHLPP ⮀ C_20_ → Akt^T^ + PHLPP (22)

where Akt^T^ and Akt^S^ denote Akt phosphorylated at Thr308 and at Ser473, respectively, and Akt^T,S^ (or Akt^S,T^) denotes the fully activated protein. C_13_ – C_20_ denote the complexes in (15)-(22).

**(VII)  *Activation/deactivation of the Akt substrates FoxO1 and GSK3β***

Upon phosphorylation by Akt^S^ and Akt^S,T^, FoxO1 leaves the nucleus and enters the cytoplasm:

FoxO1_nucl_ + Akt^S^ ⮀ C_21_ → Akt^S^ + FoxO1_cyt_ . (23)

A similar reaction holds for Akt^S,T^ with the same kinetic constants.

GSK3β is phosphorylated at Ser9 and deactivated by phosphorylated Akt, but it may also be sequestered in cytoplasmic vesicles [19]. We have

GSK3β + Akt^T^ ⮀ C_23_ → Akt^T^ + GSK3β _i_  (24)

and similarly for Akt^S,T^ with the same kinetic constants. Binding with the factor W that induces GSK3β sequestration is represented similarly.

**(VIII)  *Activation/deactivation of mTORC1 and S6K1***

Akt^T^ and Akt^T,S^ phosphorylate and inactivate the tuberous sclerosis complex 2 (TSC2). TSC2 inactivation inhibits the formation of complex Rheb/GDP and enhances the active complex Rheb/GTP, which in turn activates mTOR complex 1. mTORC1 is also inhibited by PRAS40 [20]. To simplify the model, we write :

Akt^T^ + mTORC1_i_ ⮀ C_24_ → Akt^T^ + mTORC1 (25)

where mTORC1 is directly activated by Akt^T^. A similar reaction occurs for Akt^T,S^, with the same kinetic constants. The inverse transition is regulated by a rate constant.

To achieve the full activation of S6K1, Thr229 phosphorylation by PDK1 follows Thr389 phosphorylation by mTORC1 [21]:

S6K1_i_ + mTORC1⮀ C_25_ → S6K1’ + mTORC1 (26)

S6K1’ + PDK1 ⮀ C_26_ → S6K1 + PDK1 (27)

where S6K1_i_ denotes the inactive form, S6K1’ the Thr389-phosphorylated form, and S6K1 the active form phosphorylated at both Thr229 and Thr389. The inverse transitions, S6K1’ → S6K1_i_ and S6K1 → S6K1’, are regulated by rate constants. As seen in reaction (6), the fully activated S6 kinase 1, S6K1, promotes serine phosphorylation of IRS1.

**(IX)  *Exocytosis/endocytosis of GLUT4***

Fully activated Akt and Akt^T^ phosphorylate and inactivate the Akt substrate 160 kDa protein (AS160), thus relieving from the inactive state the Rab proteins implicated in the vesicular traffic [3,22,23]. As in [1], GLUT4 also undergoes trafficking between cytoplasm and plasma membrane in basal conditions. Insulin stimulation promotes GLUT4 exocytosis according to the following reactions:

AS160 + Akt^T^ ⮀ C_28_ → Akt^T^ + AS160_i_ (28)

AS160 + Rab ⮀ C_29_ → AS160 + Rab_i_ (29)

GLUT4_cyt_ + Rab ⮀ C_30_ → Rab + GLUT4_pm_ (30)

and similarly as (28) for Akt^T,S^. The inverse reactions are regulated by rate constants, and the subscript “i” denotes the inactive form. The rate constants of exocytosis and endocytosis are *k_exo_* and, respectively, *k_endo_*.

### S2 Text. Kinetic and equilibrium equations

We discuss in detail the derivation of the kinetic and equilibrium equations for reactions (1)-(7) in Text S1, denoting concentration with the same symbol, written in italic, of the chemical species. The term of biosynthesis appears in the equation of the unmodified species. For the complexes C_0_ - C_6_ and C_P_ we write the following equations:

$$\frac{d}{dt}C_{0}=k_{0a}IR\cdot I-\left( k_{0d}+k_{0c} \right)C_{0} (1)$$

$$\frac{d}{dt}C_{1}=k_{1a}{IR}^{Y}\cdot PTP1B-\left( k_{1d}+k_{1c} \right)C_{1} (2)$$

$$\frac{d}{dt}C_{2}=k_{2a}{IR}^{Y}\cdot IRS1-\left( k_{2d}+k_{2c} \right)C_{2} (3)$$

$$\frac{d}{dt}C_{3}=k_{3a}{IRS1}^{Y}\cdot PTP1B-\left( k_{3d}+k_{3c} \right)C_{3} (4)$$

$$\frac{d}{dt}C_{4}=k_{4a}IRS1\cdot S6K1-\left( k_{4d}+k_{4c} \right)C_{4} (5)$$

$$\frac{d}{dt}C_{5}=k_{5a}{IRS1}^{S}\cdot PP-\left( k_{5d}+k_{5c} \right)C_{5} (6)$$

$$\frac{d}{dt}C_{6}^{'}=k_{6a}^{'}{IRS1}^{Y}\cdot p110_{cyt}-\left( k_{6d}^{'}+k_{6c}^{'} \right)C_{6}^{'} (7)$$

$$\frac{d}{dt}C_{6}=k_{6a}{IRS1}^{Y}\cdot p110_{PM}-\left( k_{6d}+k_{6c} \right)C_{6} (8)$$

$$\frac{d}{dt}C_{P}=k_{Pa}PTP1B\cdot pAkt-\left( k_{Pd}+k_{Pc} \right)C_{P} (9)$$

where we have assumed that the degradation rate constant of a complex enzyme-substrate is negligible compared with the sum of dissociation and catalytic constants according to the observations in [24]. An equation similar to (2) with ${IR}_{b}^{Y}$ instead of ${IR}^{Y}$ holds for $C_{1}^{'}$. In (B7)-(B8) ${IRS1}^{Y}$ denotes the concentration of complex IRS1^Y^:p85.

At the equilibrium (time derivatives equal to zero), we have

$$C_{0}=\frac{IR\cdot I}{K_{m,0}} , C_{1}=\frac{{IR}^{Y}\cdot PTP1B}{K_{m,1}} (10)$$

$$C_{2}=\frac{IRS1\cdot{IR}^{Y}}{K_{m,2}} , C_{3}=\frac{{IRS1}^{Y}\cdot PTP1B}{K_{m,3}} (11)$$

$$C_{4}=\frac{IRS1\cdot S6K1}{K_{m,4}}, C_{5}=\frac{{IRS1}^{S}\cdot PP}{K_{m,5}} . (12)$$

#### Similarly, for $C_{P}$ , $C_{6}^{'}$ and C_6_ we write

$$C_{P}=\frac{PTP1B\cdot pAkt}{K_{m,P}} , C_{6}^{'}=\frac{{IRS1}^{Y}\cdot p110_{cyt}}{K_{m,6}^{'}}, C_{6}=\frac{{IRS1}^{Y}\cdot p110_{pm}}{K_{m,6}} . (13)$$

#### The kinetics of $IR$, $IR^{Y}$, and of the spontaneously autophosphorylated IR, ${IR}_{b}^{Y}$, is described by

$$\frac{d}{dt}IR=-k_{0a}IR\cdot I+k_{0d}C_{0}+k_{1c}C_{1}+b_{IR}-\mu_{IR}IR-k_{b}IR+k_{1c}C_{1}^{'} (14)$$

$$\frac{d}{dt}{IR}^{Y}=k_{0c}C_{0}-k_{1a}{IR}^{Y}\cdot PTP1B+k_{1d}C_{1}-k_{2a}{IR}^{Y}\cdot IRS1+\left( k_{2d}+k_{2c} \right)C_{2}-\mu_{IR}{IR}^{Y} (15)$$

####

$${\frac{d}{dt}{IR}_{b}^{Y}=k}_{b}IR-k_{1a}{IR}_{b}^{Y}\cdot PTP1B+k_{1d}C_{1}^{'}-\mu_{IR}{IR}_{b}^{Y} , (16)$$

where $b_{IR}$ , expressed as concentration over time, is the rate of biosynthesis of the insulin receptor and $\mu_{IR}$ is a rate constant representing degradation. For IRS1 and PTP1B, we write:

####

$$\frac{d}{dt}IRS1=-k_{2a}IRS1\cdot{IR}^{Y}+k_{2d}C_{2}+k_{3c}C_{3}-k_{4a}IRS1\cdot S6K1+k_{4d}C_{4}+k_{5c}C_{5}$$

####

$$+b_{IRS1}-\mu_{IRS1}IRS1 (17)$$

####

$$\frac{d}{dt}{IRS1}^{Y}=k_{2c}C_{2}-k_{3a}{IRS1}^{Y}\cdot PTP1B+k_{3d}C_{3}-k_{6a}^{'}{IRS1}^{Y}\cdot p110_{cyt}+\left( k_{6d}^{'}+k_{6c}^{'} \right)C_{6}^{'}-k_{6a}{IRS1}^{Y}\cdot p110_{PM}+\left( k_{6d}+k_{6c} \right)C_{6}-\mu_{IRS1}{IRS1}^{Y} (18)$$

$$\frac{d}{dt}{IRS1}^{S}=k_{4c}C_{4}-k_{5a}{IRS1}^{S}\cdot PP+k_{5d}C_{5}-\mu_{IRS1}{IRS1}^{S} (19)$$

####

$$\frac{d}{dt}PTP1B=-k_{1a}{IR}^{Y}\cdot PTP1B+{\left( k_{1d}+k_{1c} \right)C_{1}-k_{3a}{IRS1}^{Y}\cdot PTP1B+\left( k_{3d}+k_{3c} \right)}C_{3}-k_{Pa}PTP1B\cdot pAkt+k_{Pd}C_{P}+k_{-P}PTP1B_{i}+b_{PTP1B}-\mu_{PTP1B}PTP1B (20)$$

$$\frac{d}{dt}{PTP1B}_{i}=k_{Pc}C_{P}-k_{-P}{PTP1B}_{i}-\mu_{PTP1B}{PTP1B}_{i} . (21)$$

#### At the equilibrium, in view of (1)-(3), Eqs. (14)-(16) give

$$-k_{0c}C_{0}+k_{1c}C_{1}+b_{IR}-\mu_{IR}IR-k_{b}IR+k_{1c}C_{1}^{'}=0 (22)$$

$$k_{0c}C_{0}-k_{1c}C_{1}-\mu_{IR}{IR}^{Y}=0 , (23)$$

####

$$k_{b}IR-k_{1c}C_{1}^{'}-\mu_{IR}{IR}_{b}^{Y}=0 , (24)$$

#### where the complex $C_{2}$ does not appear because its degradation rate constant has been taken equal to zero. From the sum of (22)-(24) we obtain the relation

$$IR+{IR}^{Y}+{IR}_{b}^{Y}=\frac{b_{IR}}{\mu_{IR}} .$$

The above equation and (23)-(24) are a linear system in $IR$, ${IR}^{Y}$and ${IR}_{b}^{Y}$, which is easily solved providing

$${IR}^{Y}=\frac{b_{IR}}{\mu_{IR}}\cdot\frac{\frac{k_{0c}}{K_{m,0}}I}{k_{b}+\mu_{IR}+\frac{k_{1c}PTP1B}{K_{m,1}}+\frac{k_{0c}}{K_{m,0}}I} , (25)$$

$${IR}_{b}^{Y}=\frac{b_{IR}}{\mu_{IR}}\cdot\frac{k_{b}}{k_{b}+\mu_{IR}+\frac{k_{1c}PTP1B}{K_{m,1}}+\frac{k_{0c}}{K_{m,0}}I} .$$

The expression of $IR$ is similarly found. We impose for the insulin the conservation equation

$$I_{e}=I+C_{0}+{IR}^{Y}.$$

In the above equation, $I_{e}$ is total insulin concentration, the insulin in the complex IR^Y^:IRS1 was neglected and terms in the right-hand-side refer to equal volumes. Using the expressions of $IR$and ${IR}^{Y}$, the conservation equation yields a second-order equation that gives $I$ as a function of $I_{e}$:

$$I= \frac{1}{2}\left( -\left( a_{b}+a_{0}^{''}+a_{1}^{''}PTP1B-I_{e} \right)+\sqrt{\left( a_{b}+a_{0}^{''}+a_{1}^{''}PTP1B-I_{e} \right)^{2}+4\left( a_{b}+a_{0}^{'}+a_{1}^{'}PTP1B \right)I_{e}} \right) (26)$$

with the following parameters:

$$a_{b}=k_{b}/\left( \frac{k_{0c}}{K_{m,0}} \right), a_{0}^{'}=\mu_{IR}/\left( \frac{k_{0c}}{K_{m,0}} \right) , a_{1}^{'}=\frac{k_{1c}}{K_{m,1}}/\left( \frac{k_{0c}}{K_{m,0}} \right) ,$$

$$a_{0}^{''}=a_{0}^{'}+\frac{b_{IR}}{\mu_{IR}}\frac{a_{0}^{'}}{K_{m,0}}+\frac{b_{IR}}{\mu_{IR}} , a_{1}^{''}= a_{1}^{'}+\frac{b_{IR}}{\mu_{IR}}\frac{a_{1}^{'}}{K_{m,0}} ,$$

where the choice of the positive sign in the square root is required to have $I>0$. Equation (26) allows us to express ${IR}^{Y}$and ${IR}_{b}^{Y}$ as functions of $I_{e}$. If $I_{e}=0$ and $k_{b}>0$ , only $\mathrm{IR}_{b}^{Y}$ survives and its expression shows that $k_{b}$ is likely to be much smaller than $k_{1c}PTP1B/K_{m,1}$, at least when PTP1B is not strongly inhibited. Note that the unit of $a_{b}$ and $a_{0}^{'}$ is a concentration and $a_{1}^{'}$ is nondimensional.

Proceeding similarly, Eqs. (17)-(19) at the equilibrium give

####

$$-k_{2c}C_{2}+k_{3c}C_{3}-k_{4c}C_{4}+k_{5c}C_{5}+b_{IRS1}-\mu_{IRS1}IRS1=0 (27)$$

$$k_{2c}C_{2}-k_{3c}C_{3}-\mu_{IRS1}{IRS1}^{Y}=0 (28)$$

$$k_{4c}C_{4}-k_{5c}C_{5}-\mu_{IRS1}{IRS1}^{S}=0 , (29)$$

and the sum of (27)-(29) provides the relation

$$IRS1+{IRS1}^{Y}+{IRS1}^{S}=\frac{b_{IRS1}}{\mu_{IRS1}} . (30)$$

#### Using the expressions of the complexes in (28)-(29) we obtain

$$k_{2c}\frac{{IR}^{Y}\cdot IRS1}{K_{m,2}}-\left( k_{3c}\frac{PTP1B}{K_{m,3}}+\mu_{IRS1} \right){IRS1}^{Y}=0 (31)$$

####

$$k_{4c}\frac{S6K1\cdot IRS1}{K_{m,4}}-\left( k_{5c}\frac{PP}{K_{m,5}}+\mu_{IRS1} \right){IRS1}^{S}=0 . (32)$$

#### Given IR^Y^, PTP1B, S6K1 and PP, Eqs. (30)-(32) are a linear system in IRS1, IRS1^Y^ and IRS1^S^ whose solution in IRS1^Y^ and IRS1^S^ is given by:

$$IRS1^{Y}=\frac{b_{IRS1}}{\mu_{IRS1}} \frac{\alpha\frac{k_{2c}}{K_{m,2}}{IR}^{Y}}{\alpha\beta+\beta\frac{k_{4c}}{K_{m,4}}S6K1+\alpha\frac{k_{2c}}{K_{m,2}}{IR}^{Y}} (33)$$

$$IRS1^{S}=\frac{b_{IRS1}}{\mu_{IRS1}} \frac{\beta\frac{k_{4c}}{K_{m,4}}S6K1}{\alpha\beta+\beta\frac{k_{4c}}{K_{m,4}}S6K1+\alpha\frac{k_{2c}}{K_{m,2}}{IR}^{Y}} (34)$$

with

$$\alpha=\mu_{IRS1}+\frac{k_{5c}}{K_{m,5}}PP, \beta=\mu_{IRS1}+\frac{k_{3c}}{K_{m ,3}}PTP1B .$$

At the equilibrium, from (20) and (21) we have

$$PTP1B_{i}+PTP1B=\frac{b_{PTP1B}}{\mu_{PTP1B}} ,$$

and from the last equation and the expression of *C_P_* with *pAkt* = *Akt^S,T^* (that is, the fully active, but not the partially active Akt phosphorylates PTP1B) we get

$$PTP1B=\frac{b_{PTP1B}}{\mu_{PTP1B}} \frac{1}{1+\frac{1}{\mu_{PTP1B}+k_{-P}}\cdot\frac{k_{Pc}}{K_{m,P}}Akt^{S,T}} . (35)$$

Following the same approach, and without reporting from now on the kinetic equations, we find for the active PI3K the expression

$$PI3K=\frac{b_{p110}}{\mu_{p110}} \frac{\frac{k_{6c}^{'}}{K_{m,6}^{'}}\frac{k_{6c}}{K_{m,6}}({IRS1}^{Y})^{2}}{E+F\cdot{IRS1}^{Y}+\frac{k_{6c}^{'}}{K_{m,6}^{'}}\frac{k_{6c}}{K_{m,6}}({IRS1}^{Y})^{2}} (36)$$

where

$$E=\left( \mu_{p110}+k_{-6}^{'} \right)\left( \mu_{p110}+k_{-6} \right) , F=\mu_{p110}\left( \frac{k_{6c}^{'}}{K_{m,6}^{'}}+\frac{k_{6c}}{K_{m,6}} \right)+\frac{k_{6c}^{'}}{K_{m,6}^{'}}k_{-6} .$$

According to reactions (9)-(11) in Text S1, we get

$$PI\left( 4,5 \right)P_{2}+PIP_{3}=\frac{b_{PI}}{\mu_{PI}} , PDK1_{i}+PDK1=\frac{b_{PDK1}}{\mu_{PDK1}}$$

so that

$$PIP_{3}=\frac{b_{PI}}{\mu_{PI}} \frac{\frac{k_{7c}}{K_{m,7}}PI3K}{\mu_{PI}+\frac{k_{8c}}{K_{m,8}}PTEN+\frac{k_{7c}}{K_{m,7}}PI3K} (37)$$

$$PDK1=\frac{b_{PDK1}}{\mu_{PDK1}} \frac{\frac{k_{9c}}{K_{m,9}}PIP_{3}}{\mu_{PDK1}+k_{-9}+\frac{k_{9c}}{K_{m,9}}PIP_{3}} . (38)$$

Equation (37) predicts that the concentration of the second messenger PIP_3_ increases with $PI3K$ if PTEN concentration is constant and still increases after PTEN deletion.

Turning to the upstream regulation of mTOR complex 2, we include the hypothetical signaling from PIP_3_ [20]and from the factor J [15]:

$$mTORC2=\frac{b_{mTORC2}}{\mu_{mTORC2}} \frac{\left( \mu_{mTORC2}+k_{-12} \right)\left( \frac{k_{10c}}{K_{m,10}}PIP_{3}+\frac{k_{11c}}{K_{m,11}}J \right)}{D} , (39)$$

where

$$D=\left( \mu_{mTORC2}+k_{-12} \right)\left( \mu_{mTORC2}+k_{-10}+k_{-11}+\frac{k_{10c}}{K_{m,10}}PIP_{3}+\frac{k_{11c}}{K_{m,11}}J \right)+\frac{k_{12c}}{K_{m,12}}S6K1\left( \mu_{mTORC2}+\frac{k_{10c}}{K_{m,10}}PIP_{3}+\frac{k_{11c}}{K_{m,11}}J \right) ,$$

showing how mTORC1 negatively regulates mTORC2 via S6K1.

The concentrations of Akt, Akt^T^, Akt^S^, and Akt^S,T^ at the steady state are derived from reactions (15)-(22) in Text S1 by solving a system of four linear equations, one of them given by

$$Akt+{Akt}^{T}+{Akt}^{S}+{Akt}^{S,T}=\frac{b_{Akt}}{\mu_{Akt}} .$$

We obtain the following expressions:

$${Akt}^{T}=\frac{b_{Akt}}{\mu_{Akt}}\cdot\frac{PDK1}{D_{1}}\left( \frac{k_{13c}}{K_{m,13}}\frac{k_{17c}}{K_{m,17}}PDK1\theta+\frac{k_{15c}}{K_{m,15}}\frac{k_{17c}}{K_{m,17}}mTORC2\left( \gamma-\eta\right)+\frac{k_{13c}}{K_{m,13}}\gamma\varepsilon\right) (40)$$

$${Akt}^{S}=\frac{b_{Akt}}{\mu_{Akt}}\cdot\frac{mTORC2}{D_{1}}\left( \frac{k_{15c}}{K_{m,15}}\frac{k_{19c}}{K_{m,19}}mTORC2\eta+\frac{k_{13c}}{K_{m,13}}\frac{k_{19c}}{K_{m,19}}PDK1\left( \gamma-\theta\right)+\frac{k_{15c}}{K_{m,15}}\gamma\delta\right) (41)$$

$${Akt}^{T,S}=\frac{b_{Akt}}{\mu_{Akt}}\cdot\frac{PDK1 mTORC2}{D_{1}}\left[ \frac{k_{15c}}{K_{m,15}}\frac{k_{17c}}{K_{m,17}}\left( \frac{k_{19c}}{K_{m,19}}mTORC2+\delta\right)+\frac{k_{13c}}{K_{m,13}}\frac{k_{19c}}{K_{m,19}}\left( \frac{k_{17c}}{K_{m,17}}PDK1+\varepsilon\right) \right] (42)$$

where the denominator $D_{1}$ is given by

$$D_{1}=\frac{k_{13c}}{K_{m,13}}\frac{k_{17c}}{K_{m,17}}PDK1^{2}\left( \frac{k_{19c}}{K_{m,19}}mTORC2+\theta\right)+\frac{k_{15c}}{K_{m,15}}\frac{k_{19c}}{K_{m,19}}mTORC2^{2}\left( \frac{k_{17c}}{K_{m,17}}PDK1+\eta\right)+$$

$$PDK1\cdot mTORC2\left( \frac{k_{15c}k_{17c}}{K_{m,15}K_{m,17}}\left( \gamma+\delta-\eta\right)+\frac{k_{13c}k_{19c}}{K_{m,13}K_{m,19}}\left( \gamma+\varepsilon-\theta\right)+\frac{k_{17c}k_{19c}}{K_{m,17}K_{m,19}}\left( \eta+\theta-\gamma\right) \right)$$

$$+PDK1\left( \frac{k_{13c}}{K_{m,13}}\gamma\varepsilon+\frac{k_{17c}}{K_{m,17}}\delta\theta\right)+mTORC2\left( \frac{k_{15c}}{K_{m,15}}\gamma\delta+\frac{k_{19c}}{K_{m,19}}\varepsilon\eta\right)+\gamma\delta\varepsilon$$

and the quantities $\gamma, \delta, \varepsilon,\eta,\theta$ express the activity of the phosphatases:

$$\gamma=\mu_{Akt}+\frac{k_{18c}}{K_{m,18}}PP2A+\frac{k_{20c}}{K_{m,20}}PHLPP, \delta=\mu_{Akt}+\frac{k_{14c}}{K_{m,14}}PP2A, \varepsilon=\mu_{Akt}+\frac{k_{16c}}{K_{m,16}}PHLPP,$$

$$\eta=\mu_{Akt}+\frac{k_{18c}}{K_{m,18}}PP2A, \theta=\mu_{Akt}+\frac{k_{20c}}{K_{m,20}}PHLPP .$$

Concerning the Akt substrates, we assumed that singly and doubly phosphorylated Akt molecules have the same catalytic activity. So, the cytoplasmic concentration of the Akt substrate FoxO1 has the expression

$$FoxO1_{cyt}=\frac{b_{FoxO}}{\mu_{FoxO}} \frac{\frac{k_{21c}}{K_{m,21}}\left( {Akt}^{S}+{Akt}^{T,S} \right)}{\mu_{deg}+k_{-21}+\frac{k_{21c}}{K_{m,21}}\left( {Akt}^{S}+{Akt}^{T,S} \right)} , (43)$$

and the concentration of GSK3β phosphorylated at Ser9 is obtained from reaction (24) as

$$GSK3\beta=\frac{b_{GSK3}}{\mu_{GSK3}} \frac{\frac{1}{\mu_{GSK3}+k_{-23}}\frac{k_{23c}}{K_{m,23}}\left( {Akt}^{T}+{Akt}^{T,S} \right)}{1+\frac{1}{\mu_{GSK3}+k_{-23}^{'}}\frac{k_{23c}^{'}}{K_{m,23}^{'}}W+\frac{1}{\mu_{GSK3}+k_{-23}}\frac{k_{23c}}{K_{m,23}}\left( {Akt}^{T}+{Akt}^{T,S} \right)} , (44)$$

where $W$ is the concentration of the putative factor that promotes GSK3β sequestration and decreased response [19].

For the activation of mTORC1 and S6K1, according to reactions (25)-(27) in Text S1, we have the following expressions:

$$mTORC1=\frac{b_{mTORC1}}{\mu_{mTORC1}} \frac{\frac{k_{24c}}{K_{m,24}}\left( {Akt}^{T}+{Akt}^{T,S} \right)}{\mu_{mTORC1}+k_{-24}+\frac{k_{24c}}{K_{m,24}}\left( {Akt}^{T}+{Akt}^{T,S} \right)} , (45)$$

and the reactions involving S6K1 give:

$$S6K1=\frac{b_{S6K1}}{\mu_{S6K1}} \frac{\frac{k_{26c}}{K_{m,26}}mTORC1\cdot\frac{k_{27c}}{K_{m,27}}PDK1}{D_{2}} (46)$$

where

$$D_{2}=\left( \mu_{S6K1}+k_{-26} \right)\left( \mu_{S6K1}+k_{-27} \right)+\mu_{S6K1}\frac{k_{27c}}{K_{m,27}}PDK1+\frac{k_{26c}}{K_{m,26}}mTORC1\left( \mu_{S6K1}+k_{-27}+\frac{k_{27c}}{K_{m,27}}PDK1 \right) .$$

Finally, for the concentration of GLUT4, the reactions (28)-(30) provide the following equation that gives the GLUT4 concentration at the plasma membrane in terms of ${Akt}^{T}+{Akt}^{S,T}$:

$${GLUT4}_{pm}=\frac{b_{GLUT}}{\mu_{GLUT}}\frac{\omega\rho+\left[ \omega\left( \mu_{Rab}+k_{-29} \right)+ \right]\left[ 1+\frac{k_{28c}}{\left( \mu_{AS}+k_{-28} \right)K_{m,28}}\left( {Akt}^{T}+{Akt}^{S,T} \right) \right]}{\rho+\left[ \mu_{Rab}+k_{-29}+ \right]\left[ 1+\frac{k_{28c}}{\left( \mu_{AS}+k_{-28} \right)K_{m,28}}\left( {Akt}^{T}+{Akt}^{S,T} \right) \right]} (47)$$

where

$$\omega=\frac{k_{exo}}{\left( \mu_{GLUT}+k_{exo}+k_{endo} \right)} , \rho=\frac{k_{29c}}{K_{m,29}}\frac{b_{AS}}{\mu_{AS}} ,$$

$$=\frac{k_{-29}}{\mu_{GLUT}+k_{exo}+k_{endo}}\frac{k_{30c}}{K_{m,30}} \frac{b_{Rab}}{\mu_{Rab}}$$

with $\omega$ possibly much smaller than 1. Equation (47) shows that a decrease in $\omega$ impairs GLUT4 translocation to plasma membrane. Moreover, at zero insulin, ${GLUT4}_{pm}$ may increase when AS160 expression is silenced ($\rho$ driven close to zero), according to the function of “brake” of AS160 [23].

**S3 Text. Improved activation model of Akt and mTOR complexes**

*A simplified model of Akt sub-cellular localization*

The trafficking of molecules within the cell is regulated by diffusion and active transport, and a complex mathematical treatment based on partial differential equations is required [25,26]. To give a simple model of Akt sub-cellular localization, we only consider phosphorylation at Thr308. So we have the Akt molecules in the cytosolic compartment (Akt_cyt_ and pAkt_cyt_), those located at the PM (Akt_pm_ and pAkt_pm_), and those in the nucleus (pAkt_nuc_). Considering also Ser473 Akt phosphorylation would increase the number of species from five to eleven, making the analysis more difficult and the steady-state concentrations less easily readable.

The scheme of the model is shown in Fig 4A of Main Text. PIP3 recruits PDK1 and Akt to the plasma membrane. For Akt_cyt_ we write

Akt_cyt_ + PIP3 ⮀ $C_{13}^{*}$ → Akt_pm_ + PIP3 ,

with the kinetic constants of complex $C_{13}^{*}$ defined accordingly. The reactions for Akt at the plasma membrane and in the cytosol are written according to the notations used in Text S1. At the PM, Akt is phosphorylated by PDK1 and dephosphorylated by PP2A. Transport of not yet phosphorylated Akt from PM back to cytosol is regulated by the rate constant $k_{-13}$. Phosphorylated Akt is transported to cytosol (rate constant $k_{mc}$), where it is dephosphorylated by PP2A or imported into the nucleus (rate constant $k_{cn}$). Export from the nucleus is regulated by $k_{nc}$.

We use the approach adopted in Text S2 to write the kinetic equations. The term of biosynthesis appears in the equation for $Akt_{cyt}$ and, for simplicity, the specificity constant $k_{c}/K_{m}$ is denoted as $K$ with the respective subscript. The following equations give the concentrations of the five Akt components at the steady state:

$Akt_{cyt}=\frac{b_{Akt}}{\mu_{Akt}} \frac{1}{D}\left[ \left[ \left( \mu_{Akt}+k_{-13} \right)\left( \mu_{Akt}+k_{mc}+K_{14}PP2A \right)+K_{13}PDK1\left( \mu_{Akt}+k_{mc} \right) \right] \left[ \left( \mu_{Akt}+K_{15}PP2A \right)\left( \mu_{Akt}+k_{nc} \right)+\mu_{Akt}k_{cn} \right] \right] ,$

$$Akt_{pm}=\frac{b_{Akt}}{\mu_{Akt}} \frac{1}{D} K_{13}^{*}PIP3\left( \mu_{Akt}+k_{mc}+K_{14}PP2A \right)\left[ \left( \mu_{Akt}+K_{15}PP2A \right)\left( \mu_{Akt}+k_{nc} \right)+\mu_{Akt}k_{cn} \right] ,$$

$$pAkt_{pm}=\frac{b_{Akt}}{\mu_{Akt}} \frac{1}{D} K_{13}^{*}PIP3 K_{13}PDK1\left[ \left( \mu_{Akt}+K_{15}PP2A \right)\left( \mu_{Akt}+k_{nc} \right)+\mu_{Akt}k_{cn} \right] ,$$

$$pAkt_{cyt}=\frac{b_{Akt}}{\mu_{Akt}} \frac{1}{D} K_{13}^{*}PIP3 K_{13}PDK1\left( \mu_{Akt}+k_{nc} \right)k_{mc} ,$$

$$pAkt_{nuc}=\frac{b_{Akt}}{\mu_{Akt}} \frac{1}{D} K_{13}^{*}PIP3 K_{13}PDK1 k_{mc}k_{cn} .$$

where the denominator $D$ equals the sum of the numerators in the above equations over $b_{Akt}/\mu_{Akt}$. Noting that the quantity $\mu_{Akt}$ is small compared to the kinetic constants, and assuming that $k_{nc} k_{cn}$ and $k_{mc} K_{15}PP2A$, the above equations show that the three concentrations of phosphorylated Akt tend to be equal. $Akt_{cyt}$ tends to be equal to $Akt_{pm}$ provided that $K_{13}^{*}PIP3 K_{13}PDK1$ and $k_{-13}$ is much smaller than these two quantities. Moreover $k_{mc}$ must be much larger than $K_{14}PP2A$.

*Improved modeling of mTORC1 activation*

A scheme of the improved model of mTORC1 activation is shown in Fig 4B of Main Text. Akt^T^ and Akt^T,S^ phosphorylate and inactivate the tuberous sclerosis complex 2 (TSC2) [27,28]. Active TSC2 promotes the binding of the GTPase Ras homolog enriched in brain (Rheb) to GDP and TSC2 inactivation stimulates the conversion from Rheb/GDP to the active Rheb/GTP, which activates mTORC1 [20]. mTORC1 is also inhibited by the raptor-interacting proline-rich Akt substrate 40 kDa (PRAS40) protein [29]. We may write the following reactions:

TSC2 + Akt^T^ ⮀ C_1_ → TSC2_i_ + Akt^T^ (1)

TSC2 + Rheb/GTP ⮀ C_2_ → Rheb/GDP + TSC2 (2)

Rheb/GTP + mTORC1_i_/PRAS40 ⮀ C_3_ → Rheb/GTP + mTORC1 + PRAS40 (3)

where TSC2_i_ denotes inactive TSC2, and mTORC1_i_/PRAS40 denotes the inactive mTORC1 with active PRAS40 bound to the mTORC1 component raptor. C_1_, C_2_, C_3_ denote the complexes in (1)-(3) and the kinetic constants are defined accordingly. The transition from inactive to active TSC2 is regulated by the rate constant $k_{-1}$, and from Rheb/GDP to Rheb/GTP by $k_{-2}$. A reaction similar to (1) holds for TSC2 activation by Akt^T,S^ with the same kinetic constants. In reaction (3), PRAS40 is released from mTORC1 upon activation by Rheb/GTP (Fig 4B).

Reactions (1)-(3) represent mTORC1 activation through the TSC2/Rheb axis. We write the reactions involving PRAS40 by considering that PRAS40 is phosphorylated at T246 and inactivated by Akt [29]. PRAS40 phosphorylation weakens PRAS40 association to mTORC1 and represses its inhibitory activity. For simplicity, we neglect the mTORC1-mediated PRAS40 phosphorylation at S183 and S221 by mTORC1 [30], which further increases PRAS40 inhibitory activity. We have:

PRAS40 + mTORC1 → mTORC1_i_/PRAS40 (4)

PRAS40 + Akt^T^ ⮀ C_5_ → PRAS40^T^ + Akt^T^ (5)

where (4) is a bimolecular reaction with association rate constant $k_{4}$ , and PRAS40^T^ denotes the inactive PRAS40 phosphorylated at T246. The transition from inactive to active PRAS40 is regulated by the rate constant *k_−_*_5_. A reaction similar to (5) holds for PRAS40 inactivation by Akt^T,S^ with the same kinetic constants. PRAS40 may be phosphorylated while still bound to raptor [29], but this pathway is not included in the present model.

The equations for the concentrations at the equilibrium are derived following the approach outlined in Text S2. Assuming that the term of TSC2 biosynthesis $b_{TSC}$ appears in the equation of active TSC2, we obtain from reaction (1) the following expression for TSC2 concentration:

$$TSC2=\frac{b_{TSC}}{\mu_{TSC}} \frac{1}{1+\frac{1}{\mu_{TSC}+k_{-1}}\cdot\frac{k_{1c}}{K_{m,1}}\left( Akt^{T}+Akt^{S,T} \right)} . (6)$$

where $\mu_{TSC}$ is the degradation rate constant and $TSC2_{i}=\frac{b_{TSC}}{\mu_{TSC}}-TSC2$. Similarly, assuming that the synthesis rate $b_{Rheb}$ appears in the equation of Rheb/GDP, reaction (2) provides

$$Rheb=\frac{b_{Rheb}}{\mu_{Rheb}} \frac{\frac{k_{-2}}{\mu_{Rheb}+k_{-2}}}{1+\frac{k_{-2}}{\mu_{Rheb}+k_{-2}}\cdot\frac{k_{2c}}{K_{m,2}}TSC2} , (7)$$

where $Rheb$ denotes the GTP-bound form.

Reactions (3)-(5) are used together to derive equations for mTORC1 and PRAS40. If the synthesis rate $b_{mTORC1}$ appears in the equilibrium equation of mTORC1, reactions (3)-(4) give for the concentration of mTORC1 the equation

$$mTORC1=\frac{b_{mTORC1}}{\mu_{mTORC1}} \frac{\mu_{mTORC1}+\frac{k_{3c}}{K_{m,3}}Rheb}{\mu_{mTORC1}+k_{4}PRAS40+\frac{k_{3c}}{K_{m,3}}Rheb} . (8)$$

To obtain the concentration of PRAS40, we first use the equilibrium equation for PRAS40^T^ that gives

$$PRAS40^{T}= PRAS40 \left( {Akt}^{T}+{Akt}^{T,S} \right) , (9)$$

where $=\left( k_{5c}/K_{m,5} \right)/\left( \mu_{PRAS}+k_{-5} \right)$. Using (9) and the equations for mTORC1_i_/PRAS40, PRAS40 and PRAS40^T^, the concentration of mTORC1 writes as

$$mTORC1=\frac{b_{mTORC1}-b_{PRAS}}{\mu_{mTORC1}}+ \frac{\mu_{PRAS}}{\mu_{mTORC1}} PRAS40 \left[ 1+ \left( {Akt}^{T}+{Akt}^{T,S} \right) \right] . (10)$$

Finally, using the equilibrium equations for $PRAS40$ and $PRAS40^{T}$ together with (9) and (10), a second-order equation with coefficients $a$, $b$ and $c$ for $PRAS40$ is obtained with solution

$$PRAS40=-\frac{b}{2a}+\sqrt{\left( \frac{b}{2a} \right)^{2}-\frac{c}{a}} . (11)$$

Neglecting $\mu_{mTORC1}$ compared with $\frac{k_{3c}}{K_{m,3}}Rheb$ in the expression of $c/a$, we have:

$$\frac{b}{2a}=\frac{b_{mTORC1}-b_{PRAS}}{2\mu_{PRAS}\left( 1+ \left( {Akt}^{T}+{Akt}^{T,S} \right) \right)}+\frac{}{2}Rheb , \frac{c}{a}=-\frac{b_{PRAS}}{\mu_{PRAS}} \frac{Rheb}{1+ \left( {Akt}^{T}+{Akt}^{T,S} \right)} ,$$

where $=k_{3c}/(K_{m,3}k_{4})$. We note that, as expected, $b_{PRAS}=0$ implies $PRAS40=0$. Moreover, $PRAS40$ decreases if ${Akt}^{T}+{Akt}^{T,S}$ increases and it is also regulated by $Rheb$.

Equation (8) shows that Rheb/GTP and PRAS40 regulate the overall activity of mTORC1. In normal cells, insulin signaling to Akt enhances the activity of Rheb/GTP and inhibits the activity of the inhibitor PRAS40, so both inputs regulate mTORC1 activity in the same direction. However, the relative strength of the two inputs may change, and it has been shown indeed that either regulation can overcome the effect of the other [29]. In this extended mTORC1 activation model, reactions (1)-(5) replace reaction (25) in Text S1 and equations (6)-(8) and (11) replace Eq. (45) of Text S2. The present model has several parameters more than the previous one, but it allows a representation of diverse cellular conditions.

To investigate the response of the system in different conditions, it is useful to write Eqs. (6)-(8) and (11) in a normalized form. As $k_{-2}/\left( \mu_{Rheb}+k_{-2} \right)$ is likely to be close to the unity, we have the following equations:

$$TSC2_{n}= \frac{1}{1+K_{TSC}\left( Akt_{n}^{T}+Akt_{n}^{T,S} \right)} , K_{TSC}=\frac{1}{\mu_{TSC}+k_{-1}} \frac{k_{1c}}{K_{m,1}}\frac{b_{Akt}}{\mu_{Akt}} , (12)$$

$$Rheb/GTP_{n}= \frac{1}{1+K_{Rheb} TSC2_{n}} , K_{Rheb}=\frac{k_{-2}}{\mu_{Rheb}+k_{-2}}\frac{k_{2c}}{K_{m,2}} \frac{b_{TSC}}{\mu_{TSC}} , (13)$$

$$mTORC1_{n}=\frac{K_{mTOR}^{\mu}+K_{mTOR}Rheb/GTP_{n}}{K_{mTOR}^{\mu}+PRAS40_{n}+K_{mTOR}Rheb/GTP_{n}} , (14)$$

where $K_{mTOR}^{\mu}$ and $K_{mTOR}$ are given by

$$K_{mTOR}^{\mu}=\frac{\mu_{mTORC1}}{k_{4} \frac{b_{PRAS}}{\mu_{PRAS}}} , K_{mTOR}=\frac{\frac{k_{3c}}{K_{m,3}} \frac{b_{Rheb}}{\mu_{Rheb}}}{k_{4} \frac{b_{PRAS}}{\mu_{PRAS}}} .$$

PRAS40 normalized concentration is

$${PRAS40}_{n}=-\frac{1}{2}\left[ \frac{}{1+K_{PRAS}\left( {Akt}_{n}^{T}+{Akt}_{n}^{T,S} \right)}+ K_{mTOR}Rheb_{n} \right]+\sqrt{\left( \frac{}{1+K_{PRAS}\left( {Akt}_{n}^{T}+{Akt}_{n}^{T,S} \right)}+K_{mTOR}Rheb_{n} \right)^{2}+\frac{K_{mTOR}Rheb_{n}}{1+K_{PRAS}\left( {Akt}_{n}^{T}+{Akt}_{n}^{T,S} \right)}} , (15)$$

where

$$=\frac{b_{mTORC1}-b_{PRAS}}{b_{PRAS}} , K_{PRAS}= \frac{b_{Akt}}{\mu_{Akt}} .$$

Equations (12)-(15) give the nondimensional concentrations of TSC2, Rheb/GTP, mTORC1 and PRAS40 in terms of six nondimensional parameters. The concentrations are regulated by the input concentration of phosphorylated Akt.

*Response to rapamycin of mTORC2*

Rapamycin/FKBP12 does not bind to a preformed mTOR complex 2, but it binds to newly synthesized mTOR. Therefore, the newly synthesized mTOR does not bind to rictor and mTORC2 assembly is partially or completely inhibited [31].

Equation (39) in Text S2 may still hold, but for the newly synthesized mTOR in the presence of rapamycin the following reaction may be written:

mTOR + rictor → (1 – *α* ) mTORC2_i_ + *α* mTOR + *α* rictor , (16)

where TORC2_i_ is the inactive mTORC2 (i.e., not yet activated by PIP3) and the factor *α* , that may depend upon cell type as well as rapamycin concentration, accounts for the partial or complete inhibition of mTORC2 assembly. It has been found, indeed, that long-term rapamycin treatment may cause a strong or a partial inhibition of Ser473 Akt phosphorylation, but also no response or even an increase in Ser473 Akt phosphorylation [31].

In the presence of a prolonged rapamycin treatment, the following steady-state equations may be written for mTOR and rictor concentrations according to reaction (16):

$$-\left( 1- \right)k mTOR rictor+b_{mTOR}-\mu_{mTOR}mTOR=0 , (17)$$

$$-\left( 1- \right)k mTOR rictor+b_{rictor}-\mu_{rictor}rictor=0 , (18)$$

where $k$ is the association rate constant and $[0,1]$, with $=0$ in the absence of rapamycin. The rate of mTORC2_i_ biosynthesis is given by

$$b_{mTORC2}= k\left( 1- \right) mTOR rictor (19)$$

and vanishes for $1$. From (17) and (18) we obtain a second-order equation for rictor concentration, and we get

$$rictor=\frac{1}{\mu_{rictor}}\left[ \frac{b_{rictor}-b_{mTOR}}{2}-\frac{\mu_{rictor}\mu_{mTOR}}{2k\left( 1- \right)}+\sqrt{R\left( \right)} \right] , (20)$$

$$mTOR=\frac{1}{\mu_{mTOR}}\left[ \frac{b_{mTOR}-b_{rictor}}{2}-\frac{\mu_{rictor}\mu_{mTOR}}{2k\left( 1- \right)}+\sqrt{R\left( \right)} \right] , (21)$$

where

$$R\left( \right)=[\frac{b_{mTOR}-b_{rictor}}{2}+\frac{\mu_{rictor}\mu_{mTOR}}{2k\left( 1- \right)} ]^{2}+\frac{b_{rictor}\mu_{mTOR}\mu_{rictor}}{k\left( 1- \right)} .$$

The limits for $1$ in (20) and (21) give $rictor=b_{rictor}/\mu_{rictor}$ and $mTOR=b_{mTOR}/\mu_{mTOR}$. The rate of inactive mTORC2 biosynthesis in the steady state is thus written in terms of the concentrations of mTOR and rictor and, under prolonged rapamycin treatment, it also depends on the parameter that is specific of cell type. If $=1$, the mTORC2 assembly is completely inhibited and its enzymatic activity is suppressed.

**References**

x

| 1. | Sedaghat AR, Sherman AJ, Quon M. A mathematical model of metabolic insulin signaling pathways. Am J Physiol Endocrinol Metab. 2002; 283(5): E1084–E1101. |
| --- | --- |
| 2. | Ravichandran LV, Chen H, Li Y, Quon MJ. Phosphorylation of PTP1B at Ser(50) by Akt impairs its ability to dephosphorylate the insulin receptor. Mol Endocrinol. 2001; 15(10): 1768–1780. |
| 3. | Thong FS, Dugani CB, Klip A. Turning signals on and off: GLUT4 traffic in the insulin-signaling highway. Physiology (Bethesda). 2005; 20: 271–284. |
| 4. | Hançer NJ, Qiu W, Cherella C, Li Y, Copps KD, White MF. Insulin and metabolic stress stimulate multisite serine/threonine phosphorylation of insulin receptor substrate 1 and inhibit tyrosine phosphorylation. J Biol Chem. 2014; 289(18): 12467–12484. |
| 5. | Harrington LS, Findlay GM, Gray A, Tolkacheva T, Wigfield S, Rebholz H, et al. The TSC1-2 tumor suppressor controls insulin-PI3K signaling via regulation of IRS proteins. J Cell Biol. 2004; 166(2): 213–223. |
| 6. | Saltiel AR, Kahn CR. Insulin signalling and the regulation of glucose and lipid metabolism. Nature. 2001; 414(6865): 799–806. |
| 7. | Um S, Frigerio F, Watanabe M, Picard F, Joaquin M, Sticker M, et al. Absence of S6K1 protects against age-and diet-induced obesity while enhancing insulin sensitivity. Nature. 2004; 431(7005): 200–205. |
| 8. | Dann SG, Selvaraj A, Thomas G. mTOR Complex1-S6K1 signaling: at the crossroads of obesity, diabetes and cancer. Trends Mol Med. 2007; 13(6): 252–259. |
| 9. | Fraenkel M, Ketzinel-Gilad M, Ariav Y, Pappo O, Karaca M, Castel J, et al. mTOR inhibition by rapamycin prevents beta-cell adaptation to hyperglycemia and exacerbates the metabolic state in type 2 diabetes. Diabetes. 2008; 57(4): 945–957. |
| 10. | Vivanco I, Sawyers CL. The phosphatidylinositol 3-Kinase AKT pathway in human cancer. Nat Rev Cancer. 2002; 2(7): 489–501. |
| 11. | Shisheva A. Phosphoinositides in insulin action on GLUT4 dynamics: not just PtdIns(3,4,5)P3. Am J Physiol Endocrinol Metab. 2008; 295(3): E536–E544. |
| 12. | Huang J, Manning BD. A complex interplay between Akt, TSC2 and the two mTOR complexes. Biochem Soc Trans. 2009; 37(Pt 1): 217–222. |
| 13. | Sarbassov DD, Guertin DA, Ali SM, Sabatini DM. Phosphorylation and regulation of Akt/PKB by the rictor-mTOR complex. Science. 2005; 307(5712): 1098–1101. |
| 14. | Zoncu R, Efeyan A, Sabatini DM. mTOR: from growth signal integration to cancer, diabetes and ageing. Nat Rev Mol Cell Biol. 2011; 12(1): 21–35. |
| 15. | Salinari S, Debard C, Bertuzzi A, Durand C, Zimmet P, Vidal H, et al. Jejunal proteins secreted by db/db mice or insulin-resistant humans impair the insulin signaling and determine insulin resistance. PLoS One. 2013; 8(2): p. e56258. |
| 16. | Dibble CC, Asara JM, Manning BD. Characterization of Rictor phosphorylation sites reveals direct regulation of mTOR complex 2 by S6K1. Mol Cell Biol. 2009; 29(21): 5657–5570. |
| 17. | Julien LA, Carriere A, Moreau J, Roux PP. mTORC1-activated S6K1 phosphorylates Rictor on threonine 1135 and regulates mTORC2 signaling. Mol Cell Biol. 2010; 30(4): 908–921. |
| 18. | Cybulski N, Hall MN. TOR complex 2: a signaling pathway of its own. Trends Biochem Sci. 2009; 34(12): 620–627. |
| 19. | Metcalfe C, Bienz M. Inhibition of GSK3 by Wnt signalling-two contrasting models. J Cell Sci. 2011; 124(Pt 21): 3537–3544. |
| 20. | Laplante M, Sabatini DM. mTOR signaling in growth control and disease. Cell. 2012; 149(2): 274–293. |
| 21. | Magnuson B, Ekim B, Fingar DC. Regulation and function of ribosomal protein S6 kinase (S6K) within mTOR signalling networks. Biochem J. 2012; 441(1): 1–21. |
| 22. | Karlsson HK, Zierath JR, Kane S, Krook A, Lienhard GE, Wallberg-Henriksson H. Insulin-stimulated phosphorylation of the Akt substrate AS160 is impaired in skeletal muscle of type 2 diabetic subjects. Diabetes. 2005; 54(6): 1692–1697. |
| 23. | Klip A, Sun Y, Chiu TT, Foley KP. Signal transduction meets vesicle traffic: the software and hardware of GLUT4 translocation. Am J Physiol Cell Physiol. 2014; 306(10): C879–C886. |
| 24. | Prakash S, Inobe T, Hatch AJ, Matouschek A. Substrate selection by the proteasome during degradation of protein complexes. Nat Chem Biol. 2009; 5(1): 29–36. |
| 25. | Cangiani A, Natalini R. A spatial model of cellular molecular trafficking including active transport along microtubules. J Theor Biol. 2010; 264(4): 614–625. |
| 26. | Sturrock M, Terry AJ, Xirodimas DP, Thompson AM, Chaplain MA. Spatio-temporal modelling of Hes1 and p53-Mdm2 intracellular signalling pathways. J Theor Biol. 2011; 273(1): 15–31. |
| 27. | Manning BD, Tee AR, Logsdon MN, Blenis J, Cantley LC. Identification of the tuberous sclerosis complex-2 tumor suprressor gene product tuberin as a target of the phosphoinositide 3-kinase/akt pathway. Mol Cell. 2002; 10(1): 151–162. |
| 28. | Dalle Pezze P, Sonntag AG, Thien A, Prentzell MT, Gödel M, Fischer S, et al. A dynamic network model of mTOR signaling reveals TSC-independent mTORC2 regulation. Sci Signal. 2012; 5(217): p. ra25. |
| 29. | Sancak Y, Thoreen CC, Peterson TR, Lindquist RA, Kang SA, Spooner E, et al. PRAS40 is an insulin-regulated inhibitor of the mTORC1 protein kinase. Mol Cell. 2007; 25(6): 903–915. |
| 30. | Wang L, Harris TE, Lawrence JCJ. Regulation of proline-rich Akt substrate of 40 kDa (PRAS40) function by mammalian target of rapamycin complex 1(mTORC1)-mediated phosphorylation. J Biol Chem. 2008; 283(23): 15619–15627. |
| 31. | Sarbassov DD, Ali SM, Sengupta S, Sheen JH, Hsu PP, Bagley AF, et al. Prolonged rapamycin treatment inhibits mTORC2 assembly and Akt/PKB. Mol Cell. 2006; 22(2): 159–168. |

x
