# Supplementary material for: Insulin Signaling in Insulin Resistance States and Cancer: A Modeling Analysis
Source: PLoS One. 2016 May 5;11(5):e0154415. doi: 10.1371/journal.pone.0154415 (PMC4858213; doi:10.1371/journal.pone.0154415)

Sensitivity Analysis L6 (Ctr)

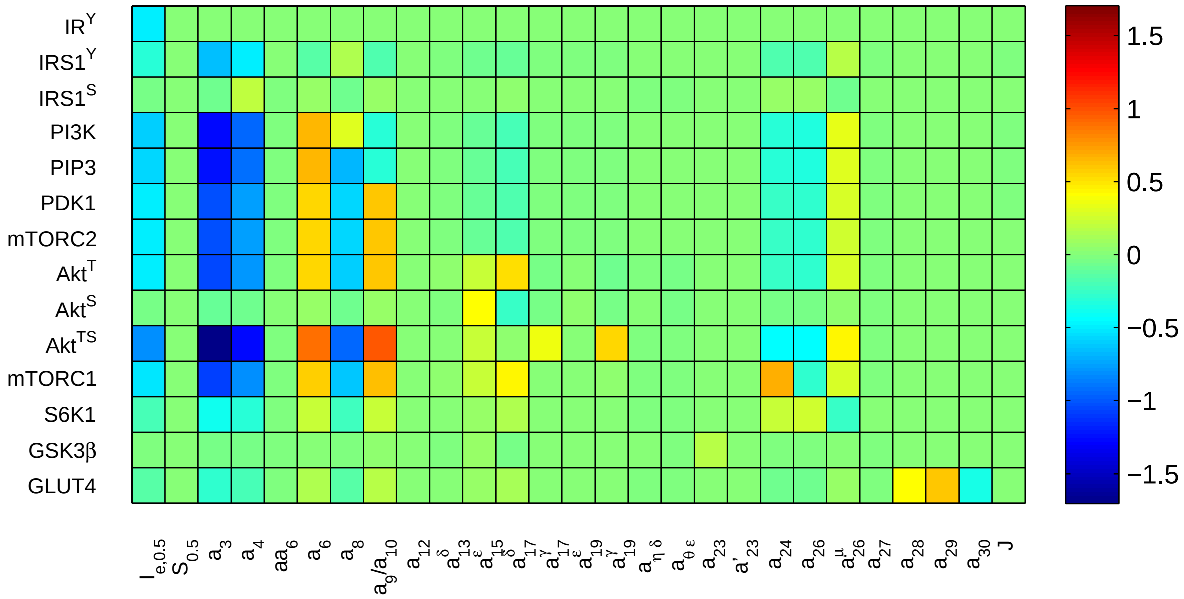

Sensitivity Analysis L6 (db/db)

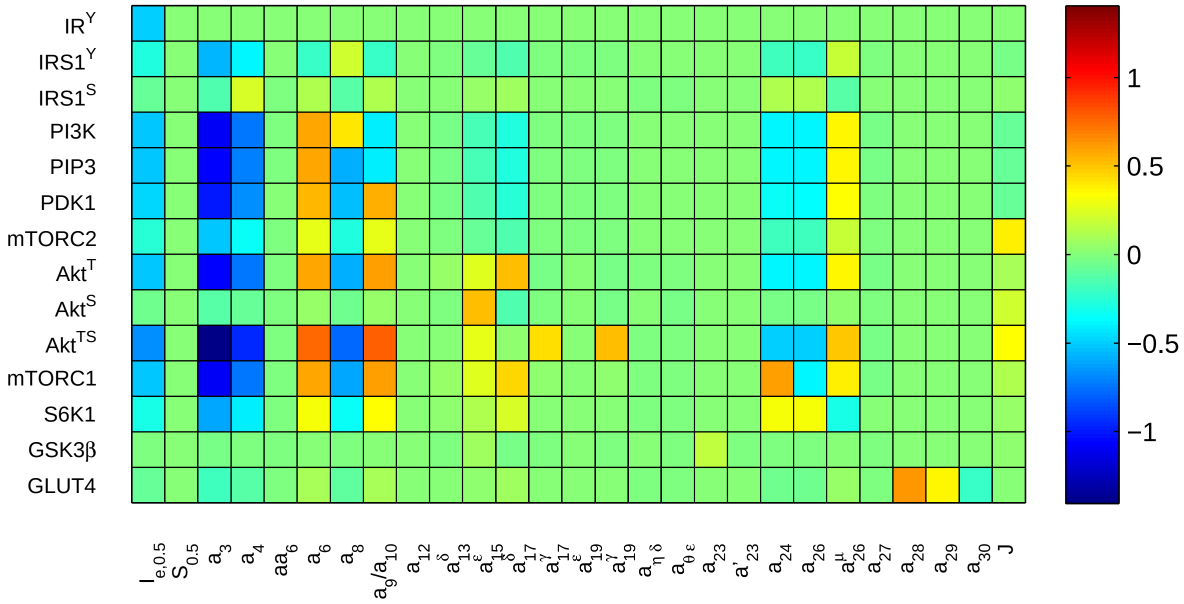

Supplement: S4 Fig — The plot shows the sensitivities of protein concentrations to the estimated parameters of the model at the extracellular insulin concentration of 9.69 nM for control cells (upper panel) and cells in db/db medium (lower panel). (PDF) [file pone.0154415.s004.pdf]
