# Supplementary material for: Insulin Signaling in Insulin Resistance States and Cancer: A Modeling Analysis
Source: PLoS One. 2016 May 5;11(5):e0154415. doi: 10.1371/journal.pone.0154415 (PMC4858213; doi:10.1371/journal.pone.0154415)
Supplement: S2 Table — (PDF) [file pone.0154415.s008.pdf]

Table S2. Parameters in Eqs. (1)-(16) in Models estimated from data of C2C12 and L6 cells.

Parameters*		C2C12	L6	
			Ctr	db/db
IR	$I_{e,0.5}\ (nM)$	44.68	9.69	–
	$S_{0.5}\ (nM^{-1})$	0.01	0.025	–
$IRS1$	a_3	8.26	1.88	–
	a_4	24.12	4.70	–
$PI3K$	a_6'	0	$1.3 \cdot 10^{-3}$	$0.66 \cdot 10^{-3}$
	a_6	124	14.50	3.62
PIP_3	a_8	0.05	1.85	–
$PDK1$	a_9	0.49	6.61	–
$mTORC2$	a_{10}	0.49	6.61	–
	a_{11}	0	1	–
	a_{12}	$5 \cdot 10^{-3}$	$1.2 \cdot 10^{-7}$	–
Akt	a_{13}^{δ}	$9.2 \cdot 10^{-4}$	0.34	–
	a_{15}^{ε}	0.22	5.80	–
	a_{17}^{δ}	0.92	3.84	–
	a_{17}^{γ}	0.20	0.29	–
	a_{19}^{ε}	7.95	0.67	–
	a_{19}^{γ}	12.88	0.65	–
	$a_{\theta\varepsilon}$	0.62	1.0	–
	$a_{\eta\delta}$	0.11	$2.5 \cdot 10^{-3}$	–
$FoxO1$**	a_{21}	0.5	0.5	–
$GSK3\beta$	a_{23}	2.92	12.54	–
	a_{23}'	0	0	3.35
$mTORC1$	a_{24}	0.01	0.17	–
$S6K1$	a_{26}	66.01	39.06	–
	a_{26}^{μ}	0.13	0.90	–
	a_{27}	74.12	460	–
$GLUT4$	a_{28}	0.14	0.22	0.15
	a_{29}	24.67	1.20	0.67
	a_{30}	28.16	2.01	–

***Parameter bounds [0, 500]. ** Parameter estimated from FoxO1 data in Figure S5 panel D.**
