# Supplementary material for: Insulin Signaling in Insulin Resistance States and Cancer: A Modeling Analysis
Source: PLoS One. 2016 May 5;11(5):e0154415. doi: 10.1371/journal.pone.0154415 (PMC4858213; doi:10.1371/journal.pone.0154415)
Supplement: S3 Table — (PDF) [file pone.0154415.s009.pdf]

Table S3. Estimates of parameters of the cell population model in control and in cells exposed to 10, 100, and 1000 nM AZD8055.

	$\alpha\,(\mathrm{hr}^{-1})$	$\mu'(\mathrm{hr}^{-1})$	$\mu''(\mathrm{hr}^{-1})$	T_2 (hr)	T_3 (hr)	$\lambda_1\,(\mathrm{hr}^{-1})$
Control	$7.43\cdot10^{-3}$	$8.52\cdot10^{-3}$	$1.81\cdot10^{-1}$	10.61	8.73	$3.4\cdot10^{-2}$
10 nM	$-1.42\cdot10^{-2}$	$1.62\cdot10^{-2}$	$1.98\cdot10^{-1}$	87.82	52.89	$3.82\cdot10^{-3}$
100 nM	$-2.12\cdot10^{-2}$	$2.16\cdot10^{-2}$	$1.1\cdot10^{-1}$	97.91	72.6	$4.56\cdot10^{-4}$
1000 nM	$-3.69\cdot10^{-2}$	$3.72\cdot10^{-2}$	$8.8\cdot10^{-2}$	97.16	77.07	$3.81\cdot10^{-4}$
