# Supplementary material for: Insulin Signaling in Insulin Resistance States and Cancer: A Modeling Analysis
Source: PLoS One. 2016 May 5;11(5):e0154415. doi: 10.1371/journal.pone.0154415 (PMC4858213; doi:10.1371/journal.pone.0154415)
Supplement: S1 Table — (PDF) [file pone.0154415.s007.pdf]

Table S1. Expression of ISN model parameters in Eqs. (1)-(16) of section Models in terms of the kinetic parameters of the equations in Text S2.

Molecule	Parameter	Expression
IR	a_b	$k_b \Big/ \left(\frac{k_{0c}}{K_{m,0}}\right)$
	a_0	$\left(\mu_{IR} + \frac{k_{1c}}{K_{m,1}} \cdot \frac{b_{PTP1B}}{\mu_{PTP1B}}\right) \Big/ \left(\frac{k_{0c}}{K_{m,0}}\right)$
	a_1	$\frac{b_{IR}}{\mu_{IR}} \cdot \frac{1}{k_{0c}} \left(k_{0c} + \mu_{IR} + \frac{k_{1c}}{K_{m,1}} \cdot \frac{b_{PTP1B}}{\mu_{PTP1B}}\right)$
$IRS1$	a_2	$\mu_{IRS1} \left(\frac{k_{2c}}{K_{m,2}} \cdot \frac{b_{IR}}{\mu_{IR}}\right)^{-1}$
	a_3	$\frac{k_{3c}}{K_{m,3}} \cdot \frac{b_{PTP1B}}{\mu_{PTP1B}} \left(\frac{k_{2c}}{K_{m,2}} \cdot \frac{b_{IR}}{\mu_{IR}}\right)^{-1}$
	a_4	$\frac{k_{4c}}{\alpha K_{m,4}} \cdot \frac{b_{S6K1}}{\mu_{S6K1}}$
$PTP1B$	a_P	$\frac{k_{Pc}}{(\mu_{PTP1B} + k_{-P})K_{m,P}} \cdot \frac{b_{Akt}}{\mu_{Akt}}$
$PI3K$	a'_6	$\frac{F}{E} \cdot \frac{b_{IRS1}}{\mu_{IRS1}}$
	a_6	$\frac{1}{E} \frac{k'_{6c}}{K'_{m,6}} \frac{k_{6c}}{K_{m,6}} \left(\frac{b_{IRS1}}{\mu_{IRS1}}\right)^2$
PIP_3	a_7	$\mu_{PI} \Big/ \left(\frac{k_{7c}}{K_{m,7}} \cdot \frac{b_{PI3K}}{\mu_{PI3K}}\right)$
	a_8	$\left(\frac{k_{8c}}{K_{m,8}} \cdot \frac{b_{PTEN}}{\mu_{PTEN}}\right) \Big/ \left(\frac{k_{7c}}{K_{m,7}} \cdot \frac{b_{PI3K}}{\mu_{PI3K}}\right)$
$PDK1$	a_9	$\frac{k_{9c}}{(\mu_{PDK1} + k_{-9})K_{m,9}} \cdot \frac{b_{PIP3}}{\mu_{PIP3}}$
$mTORC2$	a_{10}	$\frac{k_{10c}}{(k_{-10} + k_{-11})K_{m,10}} \frac{b_{PIP3}}{\mu_{PIP3}}$
	a_{10}^{μ}	$\frac{\mu_{mTORC2}}{k_{-10} + k_{-11}}$
	a_{11}	$\frac{k_{11c}}{(k_{-10} + k_{-11})K_{m,11}}$
	a_{12}	$\frac{k_{12c}}{(\mu_{mTORC2} + k_{-12})K_{m,12}} \frac{b_{S6K1}}{\mu_{S6K1}}$

Akt	a_{13}^{δ}	$\frac{k_{13c}}{K_{m,13}} \cdot \frac{b_{PDK1}}{\mu_{PDK1}} \frac{1}{\delta}$
	a_{15}^{ε}	$\frac{k_{15c}}{K_{m,15}} \cdot \frac{b_{mTORC2}}{\mu_{mTORC2}} \frac{1}{\varepsilon}$
	a_{17}^{δ}	$\frac{k_{17c}}{K_{m,17}} \cdot \frac{b_{PDK1}}{\mu_{PDK1}} \frac{1}{\delta}$
	a_{17}^{γ}	$\frac{k_{17c}}{K_{m,17}} \cdot \frac{b_{PDK1}}{\mu_{PDK1}} \frac{1}{\gamma}$
	a_{19}^{ε}	$\frac{k_{19c}}{K_{m,19}} \cdot \frac{b_{mTORC2}}{\mu_{mTORC2}} \frac{1}{\varepsilon}$
	a_{19}^{γ}	$\frac{k_{19c}}{K_{m,19}} \cdot \frac{b_{mTORC2}}{\mu_{mTORC2}} \frac{1}{\gamma}$
	$a_{\theta\varepsilon}$	θ/ε
	$a_{\eta\delta}$	η/δ
$FoxO1$	a_{21}	$\frac{1}{\mu_{deg} + k_{-21}} \cdot \frac{k_{21c}}{K_{m,21}} \frac{b_{Akt}}{\mu_{Akt}}$
$GSK3\beta$	a_{23}	$\frac{1}{\mu_{GSK3} + k_{-23}} \cdot \frac{k_{23c}}{K_{m,23}} \frac{b_{Akt}}{\mu_{Akt}}$
	a'_{23}	$\frac{1}{\mu_{GSK3} + k'_{-23}} \cdot \frac{k'_{23c}}{K'_{m,23}}$
$mTORC1$	a_{24}	$\frac{1}{\mu_{mTORC1} + k_{-24}} \cdot \frac{k_{24c}}{K_{m,24}} \frac{b_{Akt}}{\mu_{Akt}}$
$S6K1$	a_{26}	$\frac{k_{26c}/K_{m,26}}{\mu_{S6K1} + k_{-26}} \cdot \frac{b_{mTORC1}}{\mu_{mTORC1}}$
	a_{26}^{μ}	$\frac{\mu_{S6K1}}{\mu_{S6K1} + k_{-26}}$
	a_{27}	$\frac{k_{27c}/K_{m,27}}{\mu_{S6K1} + k_{-27}} \cdot \frac{b_{PDK1}}{\mu_{PDK1}}$
$GLUT4$	a_{28}	$\frac{\omega\rho + \omega(\mu_{Rab} + k_{-29}) + \sigma}{\rho + \mu_{Rab} + k_{-29} + \sigma}$
	a_{29}	$\frac{[\omega(\mu_{Rab} + k_{-29}) + \sigma]}{(\rho + \mu_{Rab} + k_{-29} + \sigma)(\mu_{AS} + k_{-28})} \cdot \frac{k_{28c}}{K_{m,28}} \cdot \frac{b_{Akt}}{\mu_{Akt}}$
	a_{30}	$\frac{\mu_{Rab} + k_{-29} + \sigma}{(\rho + \mu_{Rab} + k_{-29} + \sigma)(\mu_{AS} + k_{-28})} \cdot \frac{k_{28c}}{K_{m,28}} \cdot \frac{b_{Akt}}{\mu_{Akt}}$
